# *Colletotrichum* Species Causing Anthracnose of Rubber Trees in China

**DOI:** 10.1038/s41598-018-28166-7

**Published:** 2018-07-11

**Authors:** Xianbao Liu, Boxun Li, Jimiao Cai, Xiaolan Zheng, Yanli Feng, Guixiu Huang

**Affiliations:** Environment and Plant Protection Institute, Chinese Academy of Tropical Agricultural Sciences (CATAS), Haikou, Hainan 571101 China

**Keywords:** Phylogenetics, Fungal biology

## Abstract

Anthracnose caused by *Colletotrichum* is one of the most severe diseases of *Hevea brasiliensis*. However, research on the diversity and geographical distribution of *Colletotrichum* remains limited in China. In this study, we investigated the phylogenetic diversity of *Colletotrichum* isolates associated with symptomatic tissues of *H*.*brasiliensis* from four provinces of China (Hainan, Guangdong, Guangxi, and Yunnan). Based on multi-locus phylogenetic analyses and phenotypic characteristics, five species were distinguished, including two known species (*C*. *fructicola*, *C*. *siamense*), one novel species of *C*. *gloeosporioides* species complex (*C*. *ledongense*), and two novel species of *C*. *acutatum* species complex (*C*. *bannanense* and *C*. *australisinense*). Of these, *C*. *siamense and C*. *australisinense* have been recognized as major causative agents of anthracnose of *H*. *brasiliensis*.

## Introduction

*Hevea brasiliensis*, better known as the rubber tree, is the only species of the family Euphorbiaceae planted commercially and is the primary source of natural rubber. Natural rubber production is affected by many diseases, of which Colletotrichum leaf disease (CLD), caused by species of the genus *Colletotrichum*, is a significant problem in Southeast Asia^1^, Sri Lanka^2^, India^3^, and China^4^. The pathogen was first identified as *C*. *heveae*^5^ and then assumed to be *C*. *gloeosporioides*^6,7^. Sreenivasaprasad *et al*. reported *C*. *acutatum* from CLD lesions on *H*. *brasiliensis* in Indonesia and Sri Lanka^8^. Jayasinghe *et al*. demonstrated that the majority of strains examined from Sri Lanka belonged to *C*. *acutatum*^2^, and this species was reported in India and China as well^3,9^. Most *Colletotrichum* species isolated from rubber trees in different countries were reported *C*. *acutatum* and *C*. *gloeosporioides*. In addition, *C*. *dematium*, *C*. *crassipes*, *C*. *karstii*, and *C*. *boninense* have also been reported from infected rubber leaves, but were regarded as being of less economic importance than the above mentioned two *Colletotrichum* species^10–12^.

Sutton suggested that relationships within the genus *Colletotrichum* were unlikely to be resolved using morphology alone^13^. Modern studies demonstrated that morphological plasticity and overlapping phenotypes make traditional taxonomic criteria unreliable for the accurate delineation of *Colletotrichum* species^14,15^. The adoption and use of multi-locus phylogenetic analysis, a polyphasic approach combining the application of molecular methods with morphological and pathogenicity data, significantly changed the classification and species concepts in *Colletotrichum*^16–21^. So far, 11 species complexes of *Colletotrichum* have been identified. The *C*. *gloeosporioides* species complexes and *C*. *acutatum* species complexes among them include more members, comprise 38 species and 36 species, respectively^22^. Some species belong to endophyte that don’t cause symptoms of disease^23^. But most species are associated with plant diseases, commonly referred to as anthracnose^16,17,21,23^. These pathogens can affect a wide range of hosts. One host may be susceptible to one or more species of *Colletotrichum*^24^. On camellia, at least seven species belonging to *C*. *gloeosporioides* species complexes are associated with anthracnose^23^. On citrus, many members of *C*. *gloeosporioides* species complexes and *C*. *acutatum* species complexes were recorded as the causes of anthracnose^25,26^. On rubber tree, many cryptic and new species have been revealed, e.g., *C*. *laticiphilum*, *C*. *acutatum*, *C*. *citri*, *C*. *nymphaeae*, and *C*. *simmondsii* in the *C*. *acutatum* species complex^17,27^ and *C*. *annellatum* in the *C*. *boninense* complex^18^. However, systematic studies of the *C*. *gloeosporioides* species complex associated with rubber trees have not been conducted.

Accurate delineation of plant pathogenic fungi is critical for the establishment of quarantine regulations, screening chemicals, and resistance breeding. The aim of the present study was to investigate the *Colletotrichum* species causing anthracnose of rubber trees in China by employing large-scale sampling and isolation, and combining morphological characterization with multi-locus phylogeny.

## Results

### Multilocus-based phylogenetic analysis

We collected 62 isolates of *Colletotrichum* spp. from diseased leaves of *Hevea brasiliensis* from the main rubber tree growing regions in China, and identified them based on phylogeny and morphological characteristics. Based on the BLAST search results on the NCBI database with the ITS sequences, all *Colletotrichum* isolates in this study were preliminarily allocated to the following species complexes: 40 isolates belong to the *C*. *gloeosporioides* species complex and 22 isolates belong to *C*. *acutatum* species complex.

**Figure 1:**
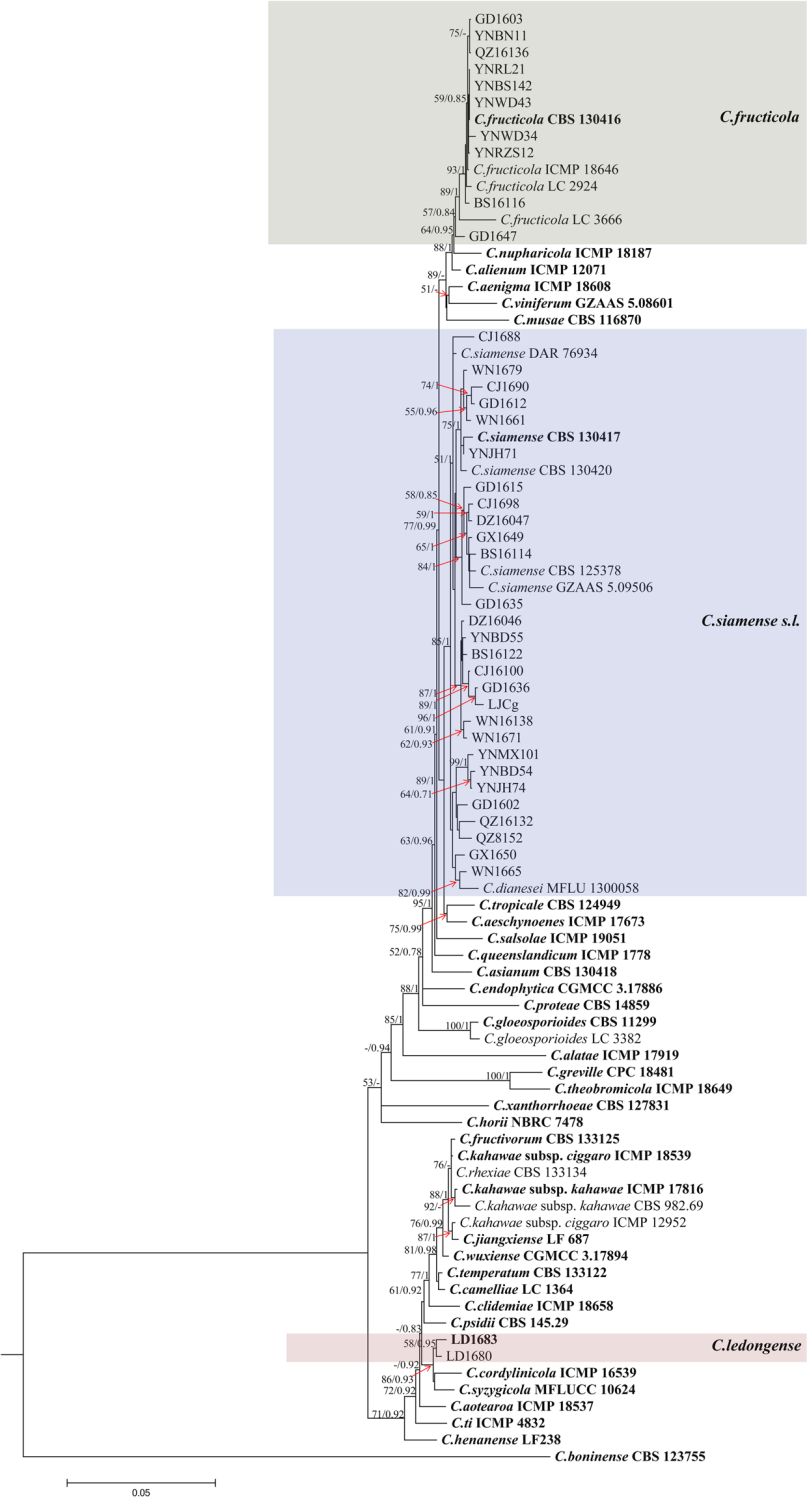
Phylogenetic tree generated by maximum likelihood analysis based on combined ITS, ACT, GAPDH, CAL, CHS-1, TUB2, and GS gene sequences. The tree displays the phylogenetic relationships between *Colletotrichum* species in the *C*. *gloeosporioides* species complex isolated from *Hevea brasiliensis*. Bootstrap support values above 50% and Bayesian posterior values above 0.7 are shown at each node (ML/PP). *C*. *boninense* CBS 123755 is used as outgroup. Ex-type strains are emphasized in bold.

The phylogram in Fig. 1 summarizes the isolates in the *C*. *gloeosporioides* species complex. The combined alignment (ITS, TUB2, CAL, ACT, GAPDH, CHS-1, and GS) contained 87 sequences, including *C*. *boninense* (CBS 123755) as an outgroup and 3,383 characters including gaps. The gene boundaries in the alignment were: ITS: 1–493, TUB2: 494–1165, CAL: 1166–1817, GAPDH: 1818–2067, ACT: 2068–2312, CHS-1: 2313–2539, GS: 2540–3383. For Bayesian analysis, The results of MrModeltest recommended a GTR + I + G model with inverse gamma distributed rate for ITS and CHS-1, a TN93 + I model for TUB2, a HKY + I model for GAPDH, a GTR + G model with gamma distributed rates for CAL and GS, a K2 + G with gamma distributed rates model for ACT. The Bayesian consensus tree (not shown) confirmed the tree topology of the maximum likelihood tree. Isolates from rubber trees in the *C*. *gloeosporioides* complex clustered in three clades (Fig. 1): ten isolates clustered with the ex-type isolates of *C*. *fructicola*, 28 isolates clustered with the ex-type isolates of *C*. *siamense*, while two isolates formed a distinct clade (posterior probability = 0.93) most closely related to *C*. *syzygicola* and *C*. *cordylinicola*.

**Figure 2:**
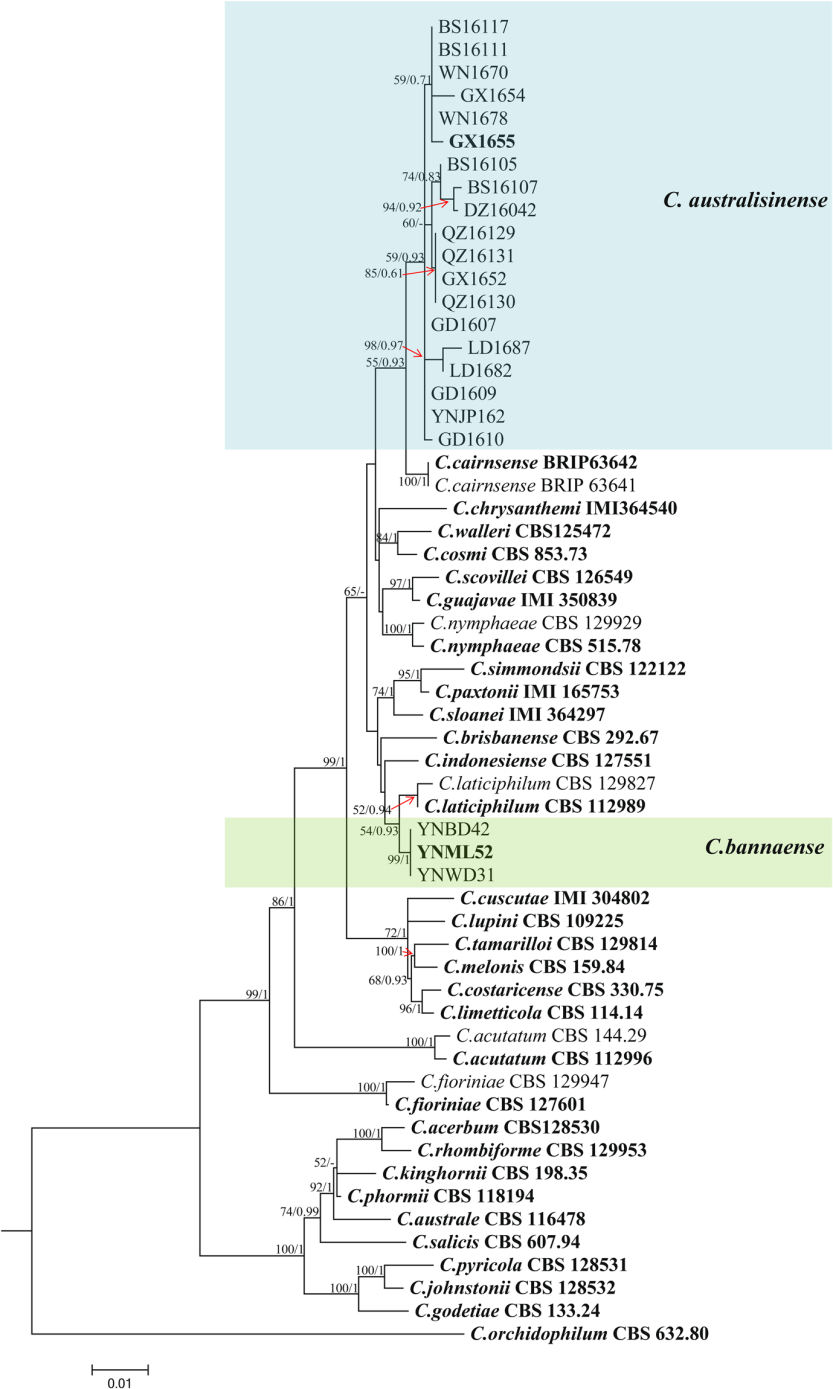
Phylogenetic tree generated by maximum likelihood analysis based on combined ITS, ACT, GAPDH, CHS-1, and TUB2 gene sequences. The tree displays the phylogenetic relationships between *Colletotrichum* species in the *C*. *acutatum* species complex isolated from *Hevea brasiliensis*. Bootstrap support values above 50% and Bayesian posterior values above 0.7 are shown at each node (ML/PP). *Colletotrichum orchidophilum* CBS 632.80 is used as outgroup. Ex-type strains are emphasized in bold.

Figure 2 demonstrates the phylogenetic relationships isolates in the *C*. *acutatum* species complex. The concatenated alignment (ITS, TUB2, ACT, GAPDH, and CHS-1) contained 58 isolates, with *C*. *orchidophilum* (CBS 632.80) as outgroup. The dataset comprised 1, 649 characters including the alignment gaps. The gene boundaries in the alignment were: ITS: 1–478, TUB: 479–961, GAPDH: 962–1197, ACT: 1198–1417, CHS-1: 1418–1649. For Bayesian analysis, The results of MrModeltest recommended a T92 + G model with gamma distributed rate for ITS, a HKY + G model with gamma distributed rates for TUB2, GAPDH, ACT. a TN93 + G model with gamma distributed rate for CHS-1. The maximum likelihood tree confirmed the tree topology and posterior probabilities of the Bayesian consensus tree. Isolates from rubber trees in the *C*. *acutatum* complex clustered in two clades (Fig. 2). Nineteen isolates formed a distinct clade (posterior probability = 0.93) most closely related to *C*. *cairnsense*. The remaining three isolates also formed a distinct clade (posterior probability = 0.93) most closely related to *C*. *laticiphilum*.

### Pairwise homoplasy index (PHI) test

**Figure 3:**
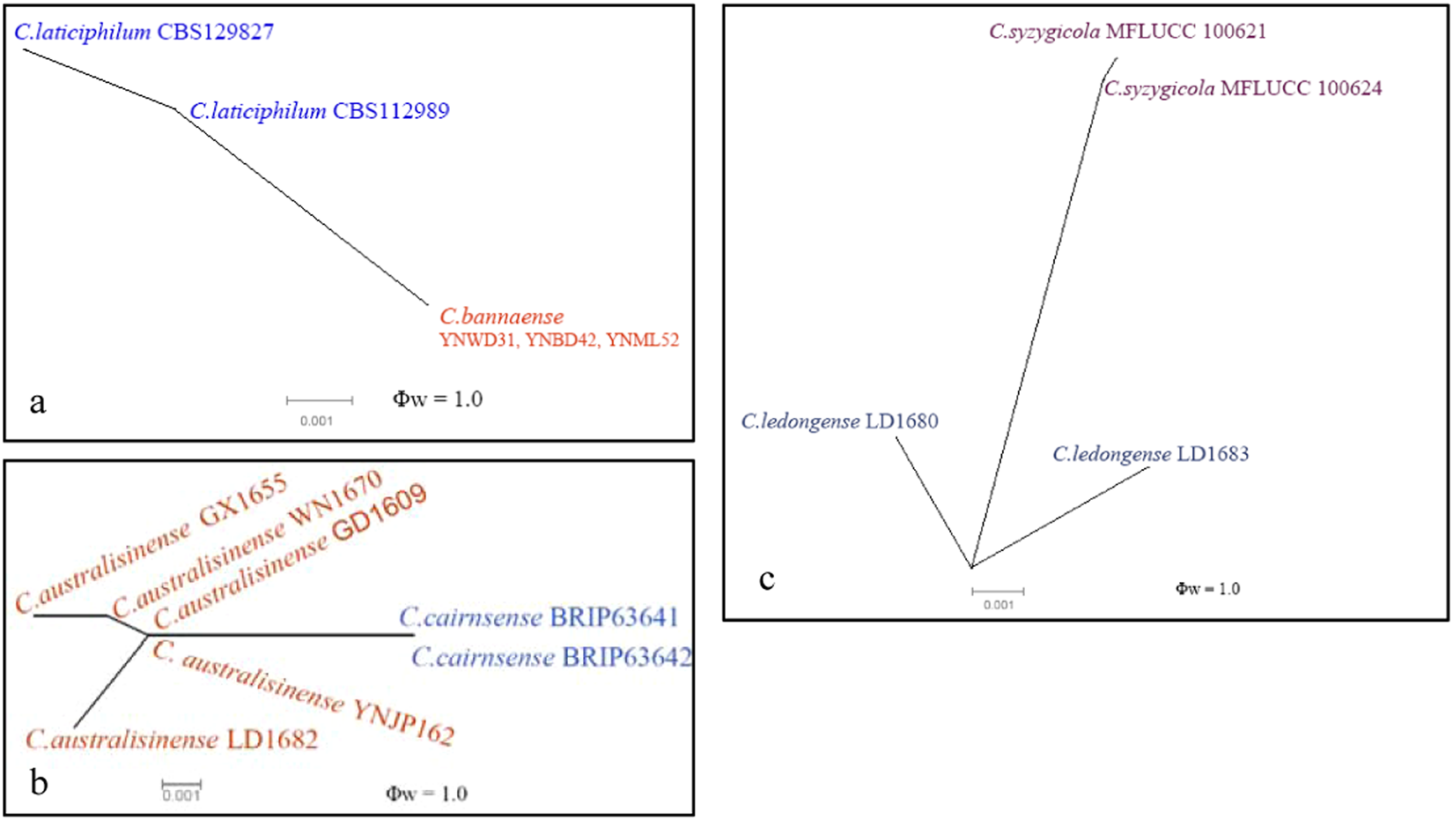
The result of the pairwise homoplasy index (PHI) test of closely related species using both LogDet transformation and splits decomposition. PHI test results (Φw) < 0.05 indicate significant recombination within the dataset.

A pairwise homoplasy index test using a 6-gene dataset (ACT, CAL, GAPDH, GS, ITS, and TUB2) was performed to determine the recombination level between *C*. *ledongense* and the closely related species *C*. *syzygicola*. No significant recombination events were detected between *C*. *ledongense* and *C*. *syzygicola* (Φw = 1) (Fig. 3).

A 5-locus concatenated dataset (ACT, GAPDH, CHS, ITS, and TUB2) was used to determine the recombination level between *C*. *bannaense* and the closely related species *C*. *laticiphilum*, as well as between *C*. *australisinense* and *C*. *cairnsense*. No significant recombination events were detected in the PHI test.

### Pathogenicity tests

**Figure 4:**
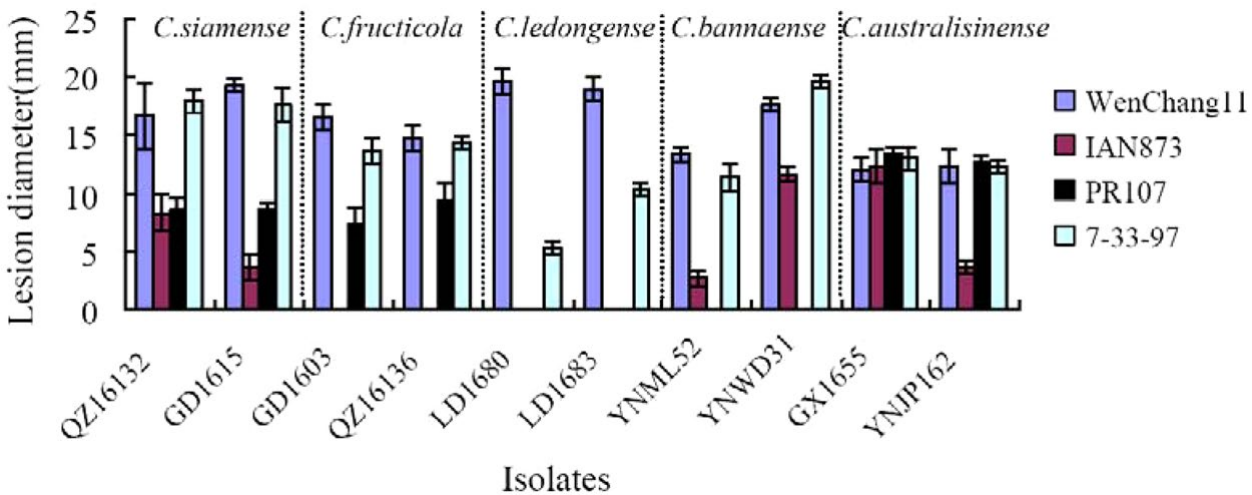
Pathogenicity of ten isolates of various *Colletotrichum* species, on four rubber tree clones (Wenchang 11, 7-33-97, IAN873, and PR107). Lesion diameter recorded 7 d post spore inoculation.

All tested isolates from symptomatic rubber tree leaves were pathogenic to rubber tree leaves. Leaves of the control plants inoculated with sterile water did not develop any symptoms 7 days post-inoculation. The inoculated *Colletotrichum* isolates could be re-isolated from the periphery of these lesions, thereby fulfilling Koch’s postulates. The pathogenicity tests (Fig. 4) demonstrated that all tested isolates were moderate or highly aggressive on Wenchang11 and 7-33-97 clone. *C*. *siamense* in the *C*. *gloeosporioides* species complex, and *C*. *australisinense* in the *C*. *acutatum* species complex were aggressive on all test rubber tree clone. However, *C*. *fructicola* was avirulent towards IAN873 clone. *C*. *ledongense* was avirulent towards both test rubber clone IAN873 and PR107, but appeared to be the highest aggressiveness on Wenchang11. *C*. *bannaense* appeared to be only weak and medium aggressive on IAN873 clone, but avirulent towards PR107. These results indicated that rubber tree Clone Wenchang11 and 7-33-97 are susceptible to *Colletotrichum* species.

### Taxonomy

Based on the multi-locus phylogeny and morphological characteristics, the 62 isolates from *H*. *brasiliensis* were identified as five species of *Colletotrichum*, including three new species and two species newly recorded from *Hevea brasiliensis* in China (*C*. *siamense* and *C*. *fructicola*).

**Figure 5:**
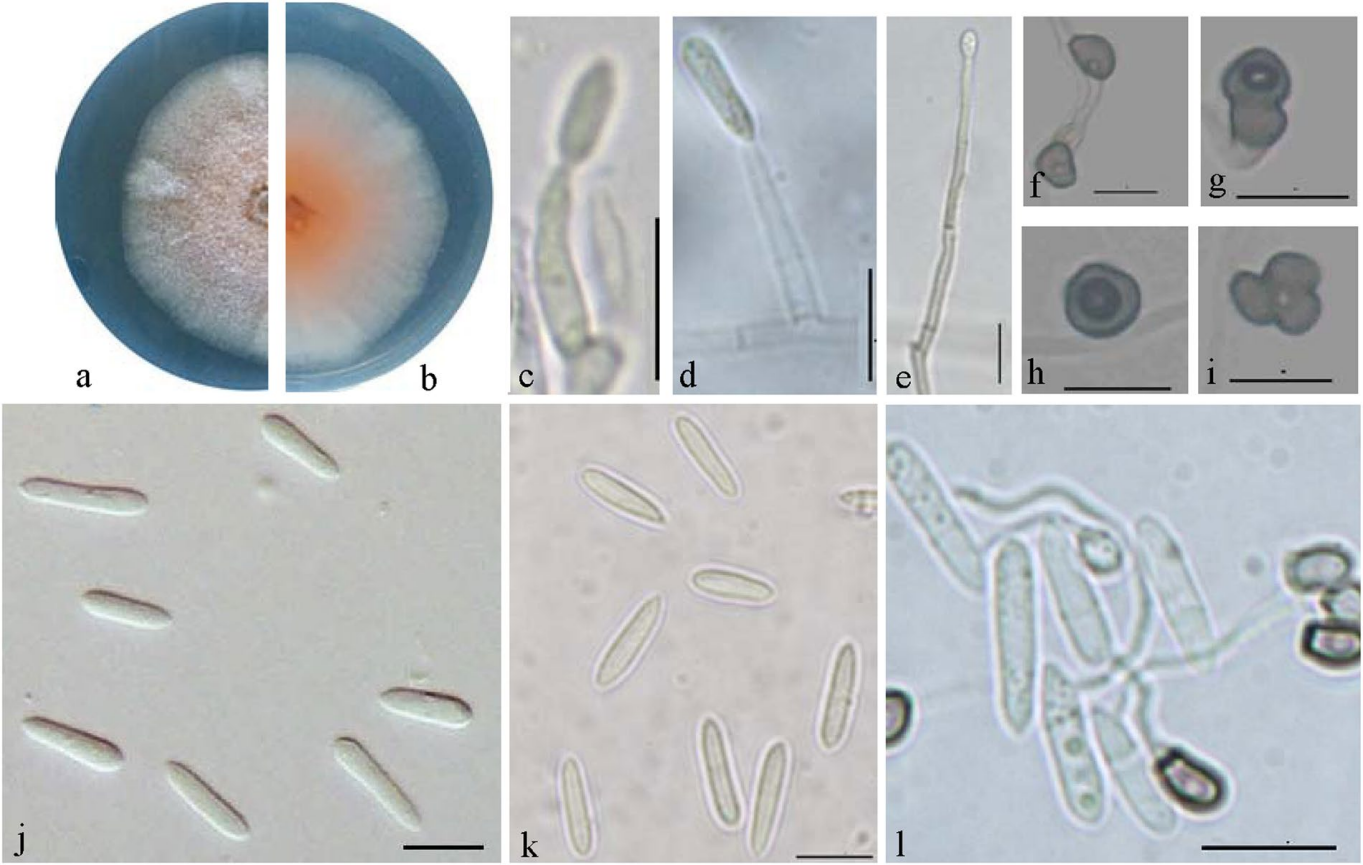
*Colletotrichum bannaense* (CGMCC 3.18887). (**a**,**b**) Forward and reverse view of culture 7 d post inoculation; (**c**–**e**) conidiophores; (**f**–**i**) appressoria (from SNA); (**j**,**k**) conidia (j from PDA; k from SNA); (**l**) Conidia germination. — Scale bar: d–i = 10 μm.

- ***Colletotrichum bannaense*** Liu X.B. **sp**. **nov**. MycoBank MB823754 Fig. 5.

### Etymology

Named after the collection site, Xishuangbanna city, Yunnan province, China.

#### On PDA

Colonies raised with entire margin, white, dense aerial mycelium; reverse white at first, then pinkish orange at the center. Colonies 40 mm diameter at 25 °C, no growth at 37 °C in 7 days. Vegetative hyphae hyaline to medium brown, usually smooth-walled, septate, and branched. Chlamydospores and setae not observed. Conidiophores either directly formed from hyphae or from a cushion of spherical hyaline cells, septate, sometimes branched. Conidiogenous cells hyaline to pale brown, cylindrical to ampulliform, straight to flexuous, 8.5–55.5 × 1.5–3 μm. Conidia hyaline, aseptate, smooth-walled, clavate to cylindrical, one end rounded and one end acute or both ends rounded, guttulate, granular, (6.5−) 10–16 (−18) × 2.5–4 μm, mean ± SD = 12.7 ± 2.8 × 3.3 ± 0.3 μm, L/W ratio = 3.8. Appressoria formed in slide culture 6.5–10.5 × 5.5–7.5 μm, mean ± SD = 8.8 ± 1.2 × 6.1 ± 0.5 μm, formed from branched mycelia, single, brown to dark brown, variable in shape, subglobose, ellipsoidal to clavate, or irregular. Sexual state not observed in culture. Conidia of other strains differ in shape and size from the ex-type strain, e.g. conidia of YNWD31 are cylindrical with both ends rounded and 9.5–16 (−20) × 2.5–5 μm, mean ± SD = 12.5 ± 1.9 × 3.6 ± 0.4 μm, L/W ratio = 3.4.

#### On SNA

Colonies flat with entire margin, hyaline. Conidiomata, chlamydospores and setae not observed. Conidiophores formed directly on hyphae or from a cushion of spherical hyaline cells. Conidia hyaline, aseptate, smooth-walled, clavate to cylindrical, one end rounded and one end acute, or both ends acute, (7−) 10–15.5 × 2–3.5 μm, mean ± SD = 12.6 ± 1.8 × 2.8 ± 0.3 μm, L/W ratio = 4.1. *Appressoria* formed in slide culture 6.5–9.5 × 5.5–7 μm, mean ± SD = 7.6 ± 1.0 × 6.0 ± 0.6 μm, single, brown to dark brown, variable in shape, subglobose, ellipsoidal to clavate, or irregular. Sexual state not observed.

### Materials examined

CHINA, Yunnan Province, Xishuangbanna city, on *Hevea brasiliensis*, 25 Sept. 2013, X.B. Liu (culture ex-type CGMCC3.18887 = YNML52); ibid., culture YNWD31, YNWD42.

**Notes —** The new species was isolated from infected rubber leaves collected from a farm in Xishuangbanna, China. This species formed a distinct clade that can be clearly distinguished from other species in the *C*. *acutatum* complex (Fig. 2). *Colletotrichum bannaense* is phylogenetically most closely related to *C*. *laticiphilum*, which was collected from *Hevea brasiliensis* in India and Colombia^17^. The sequence data of ITS, ACT, and CHS-1 do not separate the two species, but they can be distinguished by GAPDH (1 bp) and TUB (7 bp). Morphologically, *C*. *bannaense* differs from *C*. *laticiphilum* by having smaller conidia (12.7 ± 2.8 × 3.3 ± 0.3 μm vs 16.6 ± 3.1 × 3.8 ± 0.4 μm) and appressoria (7.6 ± 1.0 × 6.0 ± 0.6 μm vs 9.2 ± 2.8 × 7.2 ± 1.0 μm).

**Figure 6:**
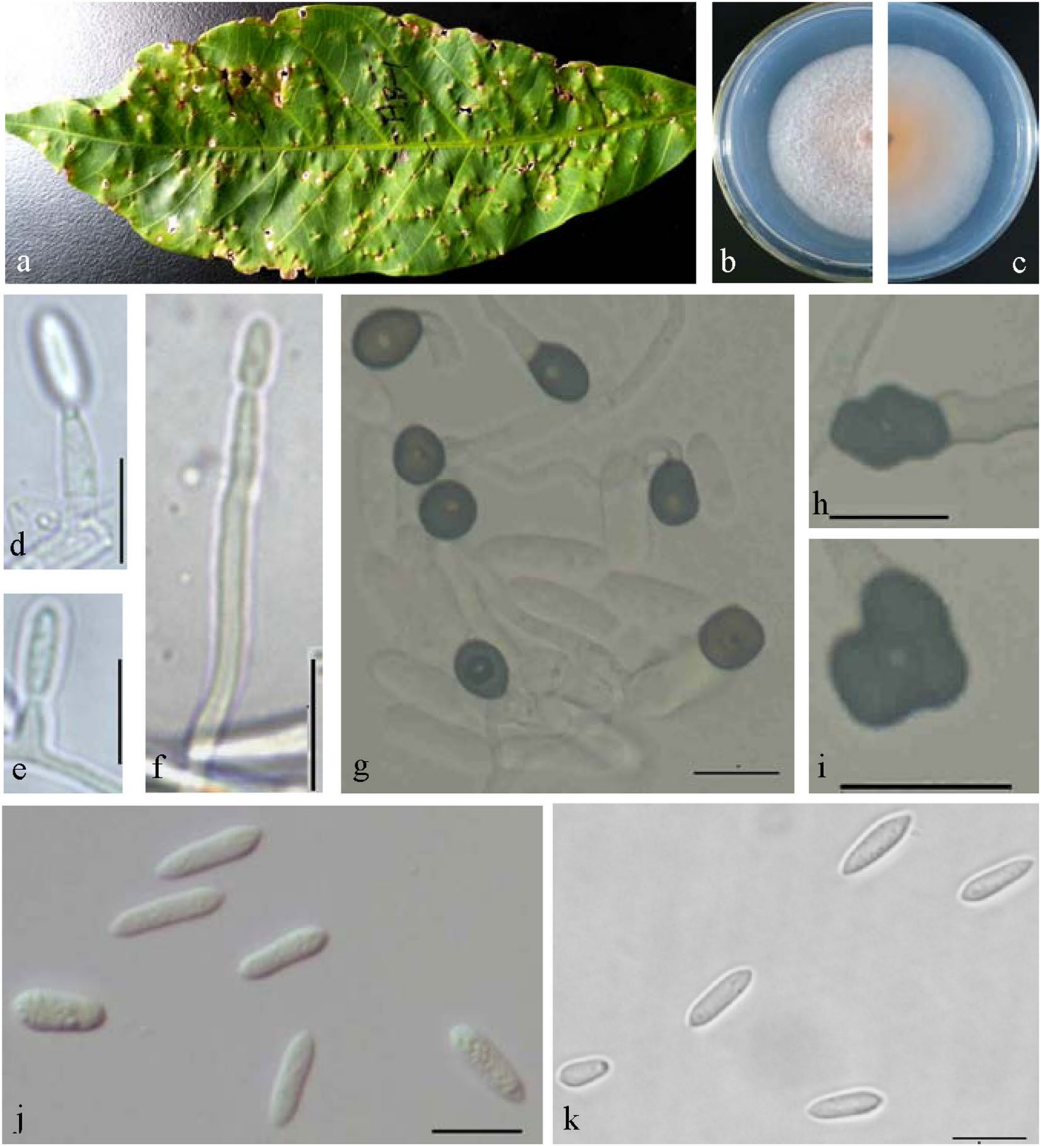
*Colletotrichum australisinense* (CGMCC 3.18886). (**a**) Symptom on rubber tree leaf; (**b**,**c**) forward and reverse view of culture 7 d post inoculation; (**d**–**f**) conidiophores; (**g**–**i**) appressoria (from SNA); (**j**,**k**) conidia (j from PDA; k from SNA). — Scale bar: d–k = 10 μm.

- ***Colletotrichum australisinense*** Liu X.B. **sp**. **nov**. MycoBank MB823813 Fig. 6.

### Etymology

Named after the geographical areas where strains were collected.

#### On PDA

Colonies raised with entire margin, white, dense aerial mycelium; reverse white at first, then orange at the center. Colonies 45 mm diameter at 25 °C and 8 mm diameter at 37 °C in 7 days. Vegetative hyphae hyaline to medium brown, usually smooth-walled, septate, branched. Conidiomata, chlamydospores, and setae absent. Conidiophores directly formed from hyphae, septate, sometimes branched. Conidiogenous cells hyaline to pale brown, cylindrical, ampulliform to conical, straight to flexuous, 9–62 × 1.5–3 μm. Conidia hyaline, aseptate, smooth-walled, cylindrical, both ends rounded or one end acute, guttulate, granular, (10.5−) 11.5–15.5 (−17.5) × 4–5.5 μm, mean ± SD = 13.4 ± 1.7 × 4.5 ± 0.4 μm, L/W ratio = 2.9. Appressoria formed in slide culture 5.5–9.5 × 5–7.5 μm, mean ± SD = 7.8 ± 1.0 × 5.9 ± 0.6 μm, formed from branched mycelia, single, brown to dark brown, variable in shape, subglobose, ovoid to ellipsoidal or irregular. Sexual state not observed.

#### On SNA

Colonies with entire margin, hyaline, sparse aerial mycelium; conidiomata not developed, chlamydospores and setae not observed. Conidia hyaline, cylindrical to fusiform, both ends acute, or one end acute, (11.5−) 12.5–18 (−19.5) × 3.5–5 μm, mean ± SD = 15.7 ± 2.3 × 4.2 ± 0.4 μm, L/W ratio = 3.7. Appressoria formed in slide culture 6.5–9.5 × 4.5–6.5 μm, mean ± SD = 7.6 ± 0.8 × 5.6 ± 0.5 μm, formed from branched mycelia, single, brown to dark brown, variable in shape, subglobose, ovoid to ellipsoidal or irregular. Sexual state not observed.

### Materials examined

CHINA, Guangxi Province, Dongxing city, on *Hevea brasiliensis*, 15 June. 2016, Liu X.B. (culture ex-type CGMCC3.18886 = GX1655); Yunnan Province, Jinping city, on *Hevea brasiliensis*, 29 Sept. 2013, culture YNJP162; Guangdong Province, Yangjiang city, on *Hevea brasiliensis*, 15 June. 2016, culture GD1609.

**Notes —***Colletotrichum australisinense* is phylogenetically most closely related to *C*. *cairnsense*, which was identified as new species from chili in Australia^28^ (Fig. 2). The sequence data of GAPDH does not separate the two species, but they can be distinguished by ITS (1 bp), ACT (1 bp), CHS-1(1 bp), and TUB (8 bp). Morphologically, *C*. *australisinense* differs from *C*. *cairnsense* by conidia shape (both ends rounded or one end acute vs two ends acute or one end slightly obtuse), having shorter conidia (13.4 ± 1.7 × 4.5 ± 0.4 μm vs 14.3 ± 0.15 × 3.7 ± 0.04 μm) on PDA, as well as by colony characters (flat, white, vs. white grey to olivaceous-grey)^28^.

**Figure 7:**
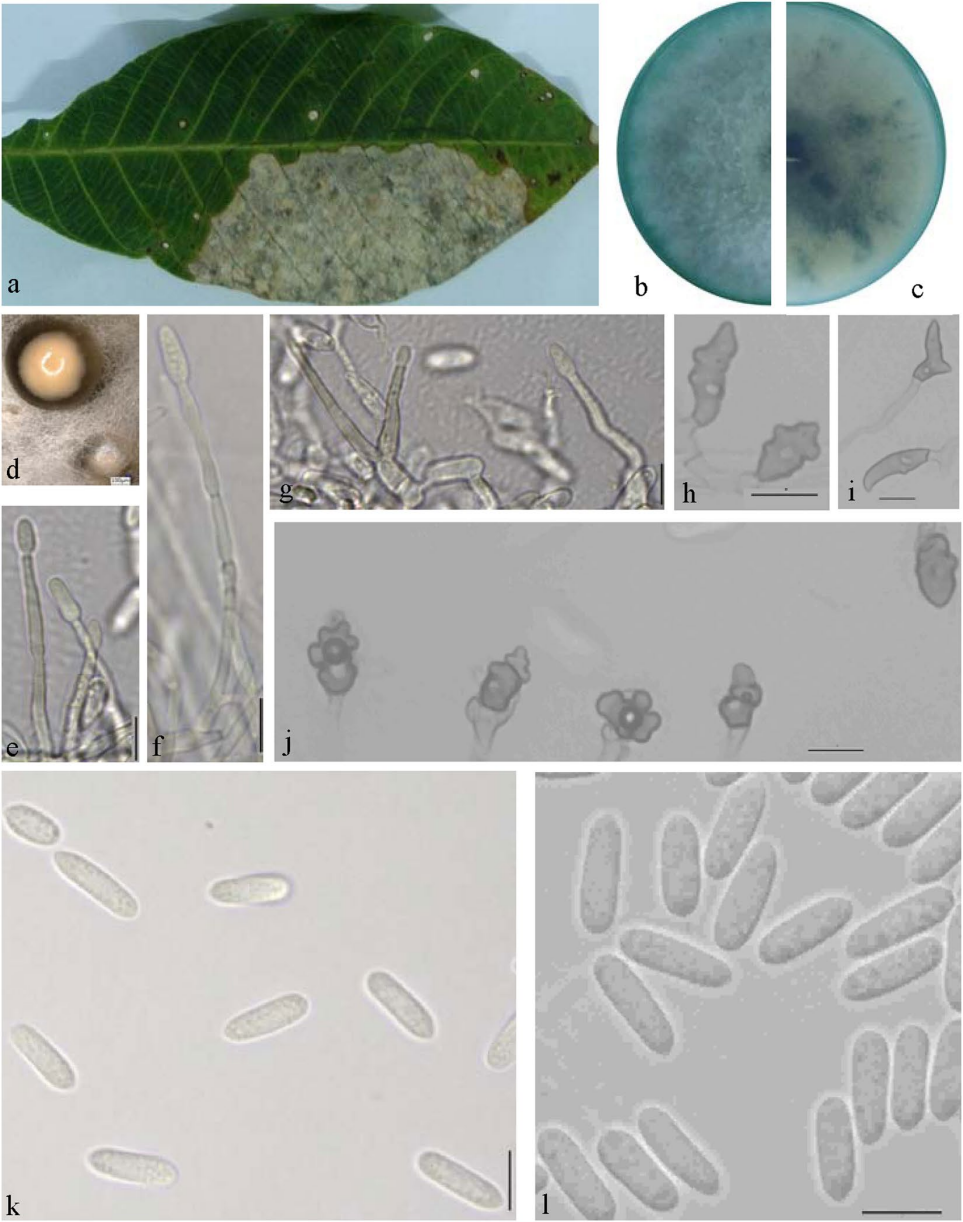
*Colletotrichum ledongense* (CGMCC 3.18888). (**a**) Symptom on rubber leaf; (**b**,**c**) forward and reverse view of culture 7 d post inoculation; (**d**) conidiomata; (**e**–**g**) conidiophores; (**h**–**j**) appressoria (SNA); (**k**,**l**) conidia; (k from PDA; l from SNA). — Scale bar: d = 100 μm; e–l = 10 μm.

- ***Colletotrichum ledongense*** Liu X.B. **sp**. **nov**. MycoBank MB823816 Fig. 7.

### Etymology

Named after the collection site, Ledong city, Hainan province, China.

#### On PDA

Colonies convex with entire margin, white to grey, dense aerial mycelium, conidiomata apricot; reverse white at first, then grey to black at the center. Colonies 75–80 mm diameter at 25 °C and 11 mm diameter at 37 °C in 7 days. Vegetative hyphae hyaline to medium brown, usually smooth-walled, septate, branched. Chlamydospores and setae not observed. Conidiophores either directly formed from hyphae or from a cushion of spherical hyaline cells, septate, sometimes branched. Conidiogenous cells hyaline to pale brown, cylindrical, straight to flexuous, 18.5–59.5 × 1.5–2.5 μm. Conidia hyaline, usually aseptate, sometimes becoming 1–2 septate with age, smooth-walled, ovoid to cylindrical or clavate, both ends rounded, or one end rounded and one end acute, guttulate, granular, (10.5−) 12.5–15 × 3.5–5.5 μm, mean ± SD = 13.8 ± 1.3 × 4.6 ± 0.5 μm, L/W ratio = 3.0. Appressoria formed in slide culture 7.5–13.5 × 6–11.5 μm, mean ± SD = 10.3 ± 2.0 × 8.1 ± 1.8 μm, formed from branched mycelia, terminal, brown to dark brown, variable in shape, irregular. Sexual state not observed in culture.

#### On SNA

Colonies flat with entire margin, hyaline, aerial mycelium sparse. Conidiomata, chlamydospores, and setae absent. Conidia hyaline, aseptate, smooth-walled, cylindrical, both ends rounded, 11.5–14.5 × 4–5 μm, mean ± SD = 12.8 ± 1.0 × 4.3 ± 0.3 μm, L/W ratio = 3.0. Appressoria formed in slide culture 9.5–15 (−18.5) × 5–8 μm, mean ± SD = 12.5 ± 2.7 × 6.5 ± 1.0 μm, formed from branched mycelia, terminal, brown to dark brown, variable in shape, irregular. Sexual state not observed.

### Materials examined

CHINA, Hainan Province, Ledong city, on *Hevea brasiliensis*, 29 Sept. 2016, Liu X.B. (culture ex-type CGMCC3.18888 = LD1683); ibid. culture LD1680.

**Notes —** The BLASTn search with the ITS sequence of LD1683 demonstrated 99% similarity to EU697202 from isolate FB2 in India, which was identified as *C*. *gloeosporioides* (Jayakumar, unpublished data). Closest matches with the TUB2 sequence was KC297088 (with 99% identity, 7 bp difference) from *C*. *aotearoa* isolate CBS 114140 from Knightia sp.^23^. Closest matches with the GAPDH sequence and the CAL sequence were KF242155 (with 99% identity, 2 bp difference) and KF254858 (with 100% identity) from the *C*. *syzygicola* strain DNCL018 from Citrus latifolia in Thailand^29^. The closest matches with the ACT sequence (100% identity)^30^ were the KC790617 from *C*. *aotearoa* strain BM2 from banana and the JX009515 *C*. *psidii* strain CBS145.29 from *Psidium* sp.^21^. The closest matches with the GS sequence (with 99% identity, 7 bp difference) were the *C*. *jiangxiense* strain LF684^31^. Based on multi-locus sequence data (ACT, CAL, GAPDH, GS, ITS, TUB2, and CHS-1), two strains of *Colletotrichum ledongense* clustered in a distinct lineage as a sister clade to *C*. *syzygicola* from *Syzygium samarangense*^29^ and *C*. *cordylinicola* from *Cordyline fruticosa*^32^. *Colletotrichum ledongense* is separated from two species by all genes except CAL, which has the same sequence as in *C*. *syzygicola*. Morphologically, *C*. *ledongense* differs from *C*. *syzygicola* and *C*. *cordylinicola* by having smaller conidia (13.8 ± 1.3 × 4.6 ± 0.5 μm vs 14.7 ± 0.9 × 5.9 ± 0.5 μm and 15.3 ± 0.6 × 4.5 ± 0.5 μm, respectively) and appressoria (12.5 ± 2.7 × 6.5 ± 1.0 μm vs 20.1 ± 1.5 × 7.6 ± 0.5 μm and 13.2 ± 0.9 × 7.2 ± 0.6 μm, respectively).

**Figure 8:**
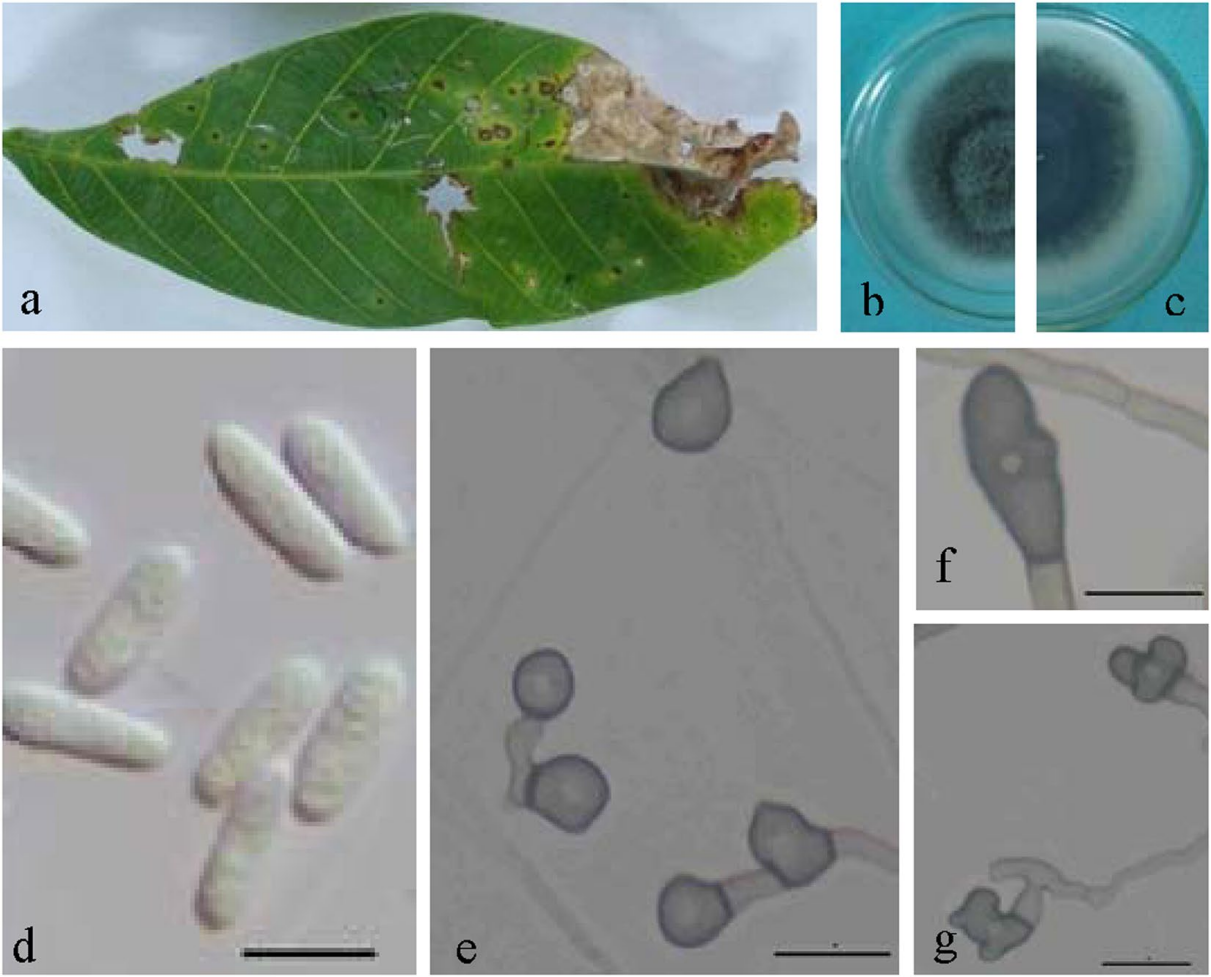
*Colletotrichum fructicola* (QZ16136). (**a**) Symptom on rubber leaf; (**b**,**c**) forward and reverse view of culture 7 d post inoculation; (**d**) conidia; (**e**–**g**) appressoria (SNA); — Scale bar: d = 100 μm; e–l = 10 μm.

- ***Colletotrichum fructicola*** Prihast., L. Cai & K.D. Hyde, Fung. Diversity 39: 158. 2009 — Fig. 8.

#### On PDA

Colonies 60–70 mm diameter at 25 °C and 14–21 mm diameter at 37 °C in 7 days. Flat with entire edge, aerial mycelium dense, cottony, grey to dark grey at the center, white at the margin, reverse greyish green with white halo. Chlamydospores and setae not observed. Conidiophores directly formed from hyphae, hyaline, septate, branched. Conidiogenous cells hyaline, cylindrical, 11–75 μm. Conidia hyaline, aseptate, smooth-walled, cylindrical, both ends rounded, or one end acute, 7–12 × 2–3.5 μm, mean ± SD = 8.9 ± 1.4 × 2.8 ± 0.3 μm, L/W ratio = 3.2. Appressoria 6–10 × 4.5–7 μm, mean ± SD = 7.7 ± 1.4 × 5.6 ± 0.9 μm, in slide cultures, formed from mycelia, brown to dark brown, solitary, circular, ovoid, clavate or irregular shapes.

### Materials examined

CHINA, Hainan Province, Qiongzhong city, on *Hevea brasiliensis*, 15 June. 2016, X.B. Liu, culture QZ16136; Yunnan Province, Xishuangbanna city, on *Hevea brasiliensis*, 29 Sept. 2013, culture YNBN11; Guangdong Province, Yangjiang city, on *Hevea brasiliensis*, 15 June. 2016, culture GD1603.

**Notes —***Colletotrichum fructicola* was primarily reported as a pathogen of coffee berries in Thailand^33^. To our knowledge, host plants for this species also include *Persea americana* (Australia), *Malus domestica* (Brazil), *Fragaria* (Canada, USA), *Limonium sp*. (Israel), *Pyrus pyrifolia* (Japan), *Dioscorea* (Nigeria), *Theobroma* and *Tetragastris* (Panama), *Ficus edulis* (Germany)^21^, *Mangifera indica* (Brazil)^34^, *Vitis* (China)^35^, *Camellia* (China)^31^, and *Corchorus capsularis* (China)^36^. However, it has not previously been reported on *Hevea brasiliensis*. In the present study, ten strains from *Hevea brasiliensis* were identified as *C*. *fructicola* based on morphology and multi-locus phylogenetic analysis. The species was found to be widely distributed throughout China. There is no significant variation in sequence data among isolates except GD1647. Conidia of isolate QZ16136 (mean ± SD = 8.9 ± 1.4 × 2.8 ± 0.3 μm) are smaller than that of the ex-type MFLU 090228 (mean ± SD = 11.53 ± 1.03 × 3.55 ± 0.32) of *C*. *fructicola*.

**Figure 9:**
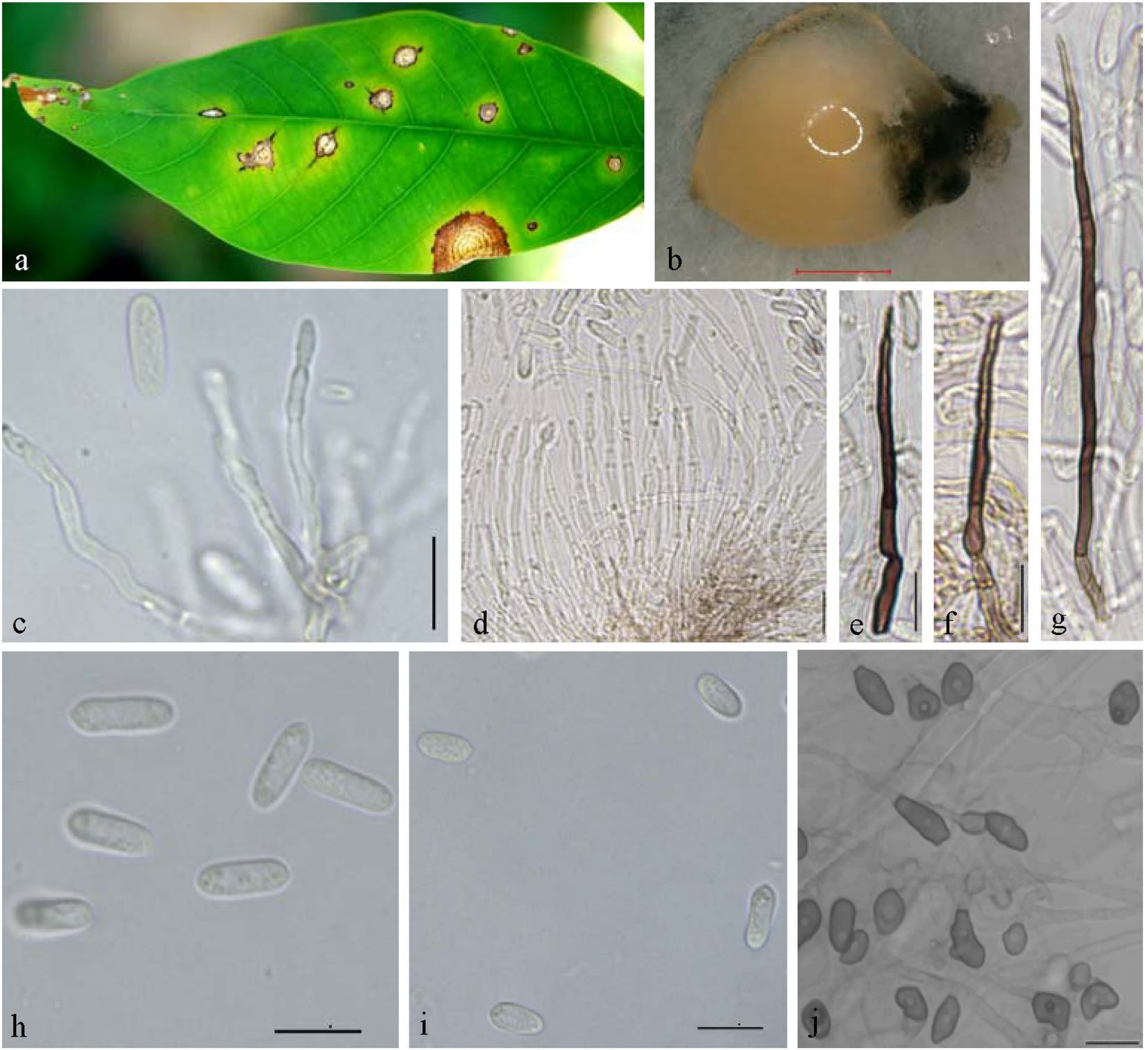
*Colletotrichum siamense* (YNBD55). (**a**) Symptom on rubber leaf; (**b**) conidiomata; (**c**,**d**) conidiophores; (**e**–**g**) seta; (**h**,**i**) conidia; (**j**) appressoria (SNA). — Scale bar: b = 500 μm; e–l = 10 μm.

- ***Colletotrichum siamense*** Prihast., L. Cai & K.D. Hyde, Fung. Diversity 39: 98. 2009 — Fig. 9.

#### On PDA

Colonies 75–85 mm diameter at 25 °C and 31–45 mm diameter at 37 °C in 7 days. Aerial mycelium white to dark-grey, cottony, surface of colony with numerous small orange conidiomata; reverse white at first, then grey to pale-olive at the center. Setae medium brown, smooth-walled, 1–3 septate, 50–87 μm long, base cylindrical, sometimes inflated, 2.5–3.5 μm diameter at its widest, tip ± acute. Chlamydospores not observed. Conidiophores formed on a cushion of medium brown roundish cells. Conidiophores hyaline, branched. Conidiogenous cells hyaline, cylindrical to ampulliform, straight to flexuous, 18–48 μm. Conidia hyaline, aseptate, smooth-walled, ovoid to cylindrical, both ends bluntly rounded, 7–13 × 2.5–4 μm, mean ± SD = 10.0 ± 1.4 × 3.6 ± 0.4 μm, L/W ratio = 2.7. Appressoria medium brown, aseptate, solitary, circular, clavate or ellipsoidal, and slightly irregular to irregular in shape, 5.5–12 × 3.5–7.5 μm, mean ± SD = 7.9 ± 1.7 × 4.4 ± 0.9 μm.

### Materials examined

CHINA, Yunnan Province, Baoding city, on *Hevea brasiliensis*, 29 Sept. 2013, X.B. Liu, culture YNBD55; Guangdong Province, Yangjiang city, on *Hevea brasiliensis*, 15 June. 2016, culture GD1615; Hainan Province, Changjiang city, on *Hevea brasiliensis*, 15 June. 2016, culture CJ1688.

**Notes** — This study supplements the morphological characteristics of setae of *Colletotrichum siamense* that were not observed in previous studies. *Colletotrichum siamense*, which was primarily reported as a pathogen of coffee berries in Thailand^33^, is a cosmopolitan pathogen causing serious diseases on many economically important plant hosts^15^. *Colletotrichum jasmini-sambac*^37^, *C*. *hymenocallidis*^38^, *C*. *dianesei*^34^, *C*. *melanocaulon*^24^, and *C*. *murrayae*^39^, which were primarily proposed as novel species closely related to *C*. *siamense*, were synonymized with *C*. *siamense* based on the Genealogical Concordance Phylogenetic Species Recognition (GCPSR) and coalescent methods by Liu *et al*.^15^. In our analysis, 28 isolates were regarded as species within *C*. *siamense* based on a multi-locus (*ACT*, *CAL*, *CHS-1*, *GAPDH*, *TUB2*, *GS*, and *ITS*) phylogenetic analysis and morphology, although they showed high sequence variability and were clustered into four clades (Fig. 1).

## Discussion

*Colletotrichum gloeosporioides* was considered as the major species responsible for Colletotrichum leaf disease of rubber trees^10,40^, until *C*. *acutarnm* was identified from CLD lesions on *Hevea brasiliensis* using ITS sequence. Jayasinghe *et al*. reported that the majority of strains examined from Sri Lanka belong to *C*. *acutatum*^2^, and Saha *et al*. reported this species from India as well. Saha *et al*. revealed that *C*. *acutatum* causes the raised spot symptom, while *C*. *gloeosporioides* causes both anthracnose and papery lesions on Hevea leaves in India^3^. In the present study, we found that the fungal pathogen responsible for CLD of rubber trees induces four different disease symptoms: the above mentioned raised spots (caused by species of the *C*. *acutatum* species complex), anthracnose, papery lesions (caused by species of the *C*. *gloeosporioides* species complex), and dark brown shrinking lesions caused by species of the *C*. *acutatum* species complex on young leaves. Referring to the CLD symptoms described by Liu *et al*. on rubber trees and the morphological character of the causal organism^4^, we suggest that *C*. *acutatum* (s. l.) was already one of the *Colletotrichum* species causing CLD on rubber trees at that time in China, and wrongly identified as *C*. *gloeosporioides*, although it was first reported in 2008^9^.

Previous studies of *Colletotrichum* species causing CLD disease of rubber trees used morphological, culture characterizations, and ITS, which restrains identification to species complexes rather than individual species^1–3,10^. *Colletotrichum gloeosporioides* and *C*. *acutatum* were identified as the two main species responsible for CLD on rubber trees. Following the use of multi-gene phylogenetic analysis, the polyphasic method for studying the genus *Colletotrichum* significantly changed the classification and species concepts. The combinations of different gene regions were recommended and delineated individual species into distinct species complexes with distinct species^16–21^. Based on multi-gene phylogenetic analysis, Damm *et al*. described new species in the *C*. *acutatum* species complexes as *Colletotrichum laticiphilum* based on TUB2, GAPDH, and CHS-1 sequences, and most differentially with TUB2^17^. Using this approach, Hunupolagama *et al*. revealed that *C*. *laticiphilum*, *C*. *acutatum*, *C*. *citri*, *C*. *nymphaeae*, and *C*. *simmondsii* belonging to the *C*. *acutatum* species complex, were causative agents of CLD on rubber trees in Sri Lanka^27^. The current study represents the first survey of *Colletotrichum* species associated with anthracnose of rubber trees in China using a multi-locus phylogenetic approach. The most striking finding of this study is the absence of the *C*. *gloeosporioides* and *C*. *acutatum* which were previously reported to be the main causal agents of rubber tree anthracnose. However, *C*. *siamense*, *C*. *fructicola*, and *C*. *ledongense*, 3 members of the *C*. *gloeosporiodes* species complex, were newly associated with rubber tree anthracnose. In addition, two new species of *C*. *acutatum* species complex, *C*. *australisinense* and *C*. *bannaense* were discovered. These newly recorded species may have been previously identified as *C*. *gloeosporioides* or *C*. *acutatum*. When comparing China with Sri Lanka, species of *C*. *acutatum* species complex on rubber trees are completely different. *Colletotrichum siamense* in the *C*. *gloeosporioides* species complex, and *C*. *australisinense* in the *C*. *acutatum* species complex are the predominant species in China, in contrast to *C*. *simmondsii* in the *C*. *acutatum* species complex in Sri Lanka.

Baroncelli *et al*. proposed the *Colletotrichum acutatum* species complex as a suitable model system to study evolution and host specialization in plant pathogens^41^. *Colletotrichum bannaense*, *C*. *australisinense*, and *C*. *laticiphilum* were found in the same host, viz., *Hevea brasiliensis*. *Colletotrichum bannaense* is most closely related to *C*. *laticiphilum* in our phylogenetic analysis. *Colletotrichum bannaense* shows limited geographical distribution and is only found in the Yunnan province of China. However, *C*. *laticiphilum* is mainly distributed in South Asia, Southeast Asia, and South America, (i.e., Sri Lanka, India, and Colombia) and shows a worldwide geographic distribution^17,27^. *Colletotrichum australisinense* is relatively distantly related to both of the above species in phylogenetic analysis, mainly distributed in Hainan, Guangdong, Guangxi, and Yunnan provinces of China. It is similar to *C*. *laticiphilum* in ITS, TUB2, ACT, and CHS-1 sequences, but there are many differences in the GAPDH sequence (15 bp/ 220 bp). The above mentioned three species are suitable for a study of gene family evolution on a fine scale to uncover evolutionary events in the genome which are associated with the evolution of phenotypic characters important for fungal plant pathogens.

Prior to the molecular era, morphological characters, such as color and growth rate of the colonies, presence or absence of setae, and size and shape of conidia and appressoria, formed the basis for studying the taxonomy of *Colletotrichum* species^7,13^. In this study, we report significant differences in growth temperatures of *Colletotrichum* species. At 37 °C, *C*. *bannaense* displayed no growth. The other four species grew, but growth rate displayed significant differences on PDA after 7 days (*C*. *siamense*, 31–45 mm; *C*. *fructicola*, 14–21 mm; *C*. *ledongense*, 9–11 mm; *C*. *australisinense*, 8 mm). We suggest that growth temperatures may be a valuable physiological criterion for the delineation of species in the genus.

## Materials and Methods

### Collection and isolates

Diseased leaves of rubber trees (*Hevea brasiliensis*) showing different symptoms (anthracnose, papery lesions, raised spot and shrinking lesions) were collected from 18 fields of four provinces in China (Hainan, Guangdong, Yunnan, and Guangxi). Plant pathogenic fungi were isolated from leaf spots using both single spore and tissue isolation methods. Single spore isolation following the protocol of Wang *et al*. was adopted for collections with visible foliar sporulation^42^. The procedure described by Niu *et al*. was used for tissue isolation and single spore cultures^36^. These pure cultures were stored in sterilized water in Eppendorf tubes at 4 °C and stock cultures were stored in PDA slants at 4 °C in the dark. The ex-type living cultures of new species from this study were deposited in the China General Microbiological Culture Collection centre (CGMCC).

### Morphological analysis

Agar plugs (5 mm diameter) were taken from the periphery of actively growing 5- days-old cultures and transferred to the center of 9 cm Petri dishes containing potato dextrose agar (PDA) and synthetic nutrient-poor agar medium (SNA). Cultures were incubated at 25 °C and 37 °C in the dark. The shapes and sizes of 30 conidia, conidiophores, and appressoria were recorded on PDA at 25 °C after 7 days using methods described by Liu *et al*.^23^. SNA was used for observation of the newly described species. Interspecific difference of colony growth was observed at 37 °C.

### DNA extraction, PCR amplification, and sequencing

Fungal isolates were grown on PDA for 7 days. Mycelia were collected in a sterile centrifuge tube and stored at −80 °C for DNA extraction. Genomic DNA was extracted from all isolates using a modified CTAB protocol as described in Stewart *et al*.^43^. In the multi-locus analysis of *C*. *gloeosporioides* species complex, seven loci, including the 5.8S nuclear ribosomal gene with the two flanking internal transcribed spacers (ITS), beta-tubulin (TUB2), calmodulin (CAL), a intron of the glyceraldehyde-3-phosphate dehydrogenase (GAPDH), a partial sequence of the actin (ACT), chitin synthase 1 (CHS-1), and glutamine synthetase (GS) gene, were amplified and sequenced using the primer pairs ITS1 + ITS4^44^, T1^45^ + Bt-2b^46^, CL1C + CL2C^21^, GDF1 + GDR1^47^, ACT-512F + ACT-783R^48^, CHS-79F + CHS-354R^48^, and GSF1^49^ + GSR2^21^, respectively. Five loci (ACT, GAPDH, ITS, TUB2, and CHS-1) were used for the multi-locus analysis of *Colletotrichum acutatum* species complex.

### Phylogenetic analysis

Sequences of ex-type and authentic strains from several contemporary phylogenetic studies^17,21,23,24,28,29,31,32,34,35,50–52^ were obtained NCBI GenBank. The dataset was assembled and manually adjusted using MEGA v. 6.0. All gaps were treated as missing data. Nucleotide substitution models were generated using MrModeltest v. 3.6^53^. A maximum likelihood phylogenetic analysis of the dataset was performed with raxmlGUI1.5^54^. Bayesian Inference analyses were performed using MrBayes v. 3.2^55^. Markov Chain Monte Carlo (MCMC) sampling was used to reconstruct phylogenies in MrBayes v. 3.2. Analyses of MCMC chains based on the full dataset were run for 1 × 10^6^ generations and sampled every 100 generations. The first 25% of the generations were discarded as burn-in. Figures of trees were created in MEGA v. 6.0. or FigTree v 1.3.1^56^. Sequences derived in this study were deposited in GenBank (Supplementary file), and taxonomic novelties in MycoBank (www.MycoBank.org)^57^.

### Genealogical concordance phylogenetic species recognition analysis

Phylogenetically related but ambiguous species were analyzed using the Genealogical Concordance Phylogenetic Species Recognition (GCPSR) model by performing a pairwise homoplasy index (PHI) test as described by Quaedvlieg *et al*.^58^. The PHI test was performed in SplitsTree4^59,60^ in order to determine the recombination level within phylogenetically closely related species using a 6-locus concatenated dataset (ACT, CAL, GAPDH, GS, ITS, and TUB2) in *C*. *gloeosporioides* species complex, and using a 5-locus concatenated dataset (ACT, GAPDH, CHS, ITS, and TUB2) in the *C*. *acutatum* species complex. If the pairwise homoplasy index results were below a 0.05 threshold (Фw < 0.05), it was indicative for significant recombination present in the dataset. The relationship between closely related species was visualized by constructing a splits graph.

### Pathogenicity

All strains were selected for pathogenicity testing: Healthy and non-wounded copper brown leaves, collected from the clone Wenchang11, 7-33-97, IAN873, and PR107, were washed with tap water and disinfected in 1% sodium hypochlorite for 3 min. Disinfected leaves were washed three times with sterilized water and then dried on the bench top. Fungal conidia were harvested by flooding 4-day-old single conidial cultures with sterile water, centrifuging, and adjusting the concentration to 1 × 10^6^ conidia/ml. Then, 20 μL conidial suspensions were dropped onto intact, detached copper brown rubber tree leaves. Leaves inoculated with sterile water were used as controls. Inoculated leaves placed on moist tissue paper were maintained in a humidified chamber, incubated at 28 °C, and monitored daily for lesion development. Finally, conidia of each strain were collected from diseased leaves and cultured on a new PDA plate. These were then checked for morphological characteristics to confirm Koch’s postulates.

## Electronic supplementary material

Supplementary Information
