# Author Correction: *Colletotrichum* Species Causing Anthracnose of Rubber Trees in China

**DOI:** 10.1038/s41598-021-96671-3

**Published:** 2021-09-06

**Authors:** Xianbao Liu, Boxun Li, Jimiao Cai, Xiaolan Zheng, Yanli Feng, Guixiu Huang

**Affiliations:** Environment and Plant Protection Institute, Chinese Academy of Tropical Agricultural Sciences (CATAS), Haikou, 571101 Hainan China

Correction to: *Scientific Reports* 10.1038/s41598-018-28166-7, published online 11 July 2018

The original version of this Article contains errors.

It was not indicated in which fungarium or culture collection the holotype material of the new species was deposited. As a consequence, the proposed new species are invalid due to Art. 40.1 of the Shenzhen Code (Turland et al. 2018)^1^.

The species are validated herein.

***Colletotrichum australisinense*** X.B. Liu, sp. nov. MycoBank MB838002

**Type**: China, Guangxi province, Dongxing, infected rubber leaves, Jun. 2016, X.B. Liu, CGMCC3.18886 (preserved in a metabolically inactive state in the China General Microbiological Culture Collection centre, i.e. CGMCC).

***Colletotrichum bannaense*** X.B. Liu, sp. nov. MycoBank MB838090

**Type**: China, Yunnan province, Xishuangbanna, infected rubber leaves, Sep. 2013, X.B. Liu, CGMCC3.18887 (preserved in a metabolically inactive state in the China General Microbiological Culture Collection centre, i.e. CGMCC).

***Colletotrichum ledongense*** X.B. Liu, sp. nov. MycoBank MB838003

**Type:** China, Hainan province, Ledong, infected rubber leaves, Sep. 2016, X.B. Liu, CGMCC3.18888 (preserved in a metabolically inactive state in the China General Microbiological Culture Collection centre, i.e. CGMCC).

For the full description of each species, please see the original Article.

## References

[1] Turland, N. J. *et al.**International Code of Nomenclature for Algae, Fungi, and Plants (Shenzhen Code) Adopted by the Nineteenth International Botanical Congress Shenzhen, China, July 2017.* Regnum Vegetabile No. 159 (Koeltz Botanical Books, 2018). 10.12705/Code.2018.

