# Supplementary material for: Colletotrichum Species Causing Anthracnose of Rubber Trees in China
Source: Sci Rep. 2018 Jul 11;8:10435. doi: 10.1038/s41598-018-28166-7 (PMC6041288; doi:10.1038/s41598-018-28166-7)
Supplement: Supplementary file 1 — Supplementary Information [file 41598_2018_28166_MOESM1_ESM.doc]

SUPPLEMENTARY INFORMATION

***Colletotrichum*** **Species Causing Anthracnose of Rubber Trees in China**

Xianbao Liu, Boxun Li, Jimiao Cai, Xiaolan Zheng, Yanli Feng,Guixiu Huang*

Environment and Plant Protection Institute, Chinese Academy of Tropical Agricultural Sciences (CATAS), Haikou, Hainan 571101, China.

*Corresponding author e-mail: [hgxiu@vip.163.com](mailto:hgxiu@vip.163.com)

**Table S 1.** Strains of the *C. gloeosporioides* s.l. species studied in this report with details on location, host, GenBank accessions of the sequences generated, and reference.

| **Species** | **Strains** | **Locality** | **Host** | **GenBank accessions** | | | | | | | Reference  **reference** |
| --- | --- | --- | --- | --- | --- | --- | --- | --- | --- | --- | --- |
|  |  |  |  | **ITS** | **TUB2** | **CAL** | **ACT** | **GAPDH** | **CHS-1** | **GS** |  |
| ***C. aenigma*** | ICMP18608* | Israel | *Persea americana* | JX010244 | JX010389 | JX009683 | JX009443 | JX010044 | JX009774 | JX010078 | Weir et al.2012 |
| ***C. aeschynomenes*** | ICMP 17673* | USA | *Aeschynomene virginica* | JX010176 | JX010392 | JX009721 | JX009483 | JX009930 | JX009799 | JX010081 | Weir et al. 2012 |
| ***C. alatae*** | ICMP 17919* | India | *Dioscorea alata* | JX010190 | JX010383 | JX009738 | JX009471 | JX009990 | JX009837 | JX010065 | Weir et al. 2012 |
| ***C. alienum*** | ICMP 12071* | New Zealand | *Malus domestica* | JX010251 | JX010411 | JX009654 | JX009572 | JX010028 | JX009882 | JX010101 | Weir et al. 2012 |
| ***C. aotearoa*** | ICMP 18537* | New Zealand | *Coprosma sp.* | JX010205 | JX010420 | JX009611 | JX009564 | JX010005 | JX009853 | JX010113 | Weir et al. 2012 |
| ***C. asianum*** | ICMP 18580* | Thailand | *Coffea arabica* | FJ972612 | JX010406 | FJ917506 | JX009584 | JX010053 | JX009867 | JX010096 | Weir et al. 2012 |
| ***C. boninense*** | MAFF 305972* | Japan | *Crinum asiaticum var.* | JQ005153 | JQ005588 | JQ005674 | JQ005501 | JQ005240 | JX009827 | — | Liu et al. 2013 |
| ***C. camelliae*** | LC1364* | China | *Ca. sinensis* | KJ955081 | KJ955230 | KJ954634 | KJ954363 | KJ954782 | — | KJ954932 | Liu et al. 2015 |
| ***C. clidemiae*** | ICMP 18658* | USA | *Clidemia hirta* | JX010265 | JX010438 | JX009645 | JX009537 | JX009989 | JX009877 | JX010129 | Weir et al. 2012 |
| ***C. cordylinicola*** | ICMP 18579* | Thailand | *Cordyline fruticosa* | JX010226 | JX010440 | HM470238 | HM470235 | JX009975 | JX009864 | JX010122 | Weir et al. 2012 |
| ***C. dianesei*** | MFLU 1300058* | Brazil | *Mangifera indica* | KC329779 | KC517254 | KC517209 | KC517298 | KC517194 | — | KC430894 | Lima et al. 2013 |
| ***C. endophytica*** | CGMCC3.17886, LC0324* | Thailand | *Pennisetum purpureum* | KC633854 | — | KC810018 | KF306258 | KC832854 | — | — | Manamgoda et al. 2013 |
| ***C. fructicola*** | CBS 130416* | Thailand | *Coffea arabica* | JX010165 | JX010405 | FJ917508 | FJ907426 | JX010033 | JX009866 | JX010095 | Weir et al. 2012 |
|  | ICMP 18646 | Panama | *Tetragastris panamensis* | JX010173 | JX010409 | JX009674 | JX009581 | JX010032 | JX009874 | JX010099 | Weir et al. 2012 |
|  | LC3666 | Indonesia | *Ca. sinensis* | KJ955221 | KJ955366 | KJ954772 | KJ954489 | KJ954920 | — | KJ955071 | Liu et al. 2015 |
|  | LC2924 | China | *Ca. sinensis* | KJ955084 | KJ955233 | KJ954637 | KJ954366 | KJ954785 |  | KJ954635 | Liu et al. 2015 |
|  | **GD1603** | **China:Guangdong** | ***H.brasiliensis*** | **MG253872** | **MG253882** | **MG253892** | **MG253902** | **MG266127** | **MG266137** | **MG266147** | This study |
|  | **YNBN11** | **China:Yunnan** | ***H.brasiliensis*** | **MG253873** | **MG253883** | **MG253893** | **MG253903** | **MG266128** | **MG266138** | **MG266148** | This study |
|  | **QZ16136** | **China:Hainan** | ***H.brasiliensis*** | **MG253874** | **MG253884** | **MG253894** | **MG253904** | **MG266129** | **MG266139** | **MG266149** | This study |
|  | **YNRL21** | **China:Yunnan** | ***H.brasiliensis*** | **MG253875** | **MG253885** | **MG253895** | **MG253905** | **MG266130** | **MG266140** | **MG266150** | This study |
|  | **YNBS142** | **China: Yunnan** | ***H.brasiliensis*** | **MG253876** | **MG253886** | **MG253896** | **MG253906** | **MG266131** | **MG266141** | **MG266151** | This study |
|  | **YNWD43** | **China: Yunnan** | ***H.brasiliensis*** | **MG253877** | **MG253887** | **MG253897** | **MG253907** | **MG266132** | **MG266142** | **MG266152** | This study |
|  | **YNWD34** | **China: Yunnan** | ***H.brasiliensis*** | **MG253878** | **MG253888** | **MG253898** | **MG253908** | **MG266133** | **MG266143** | **MG266153** | This study |
|  | **YNRZS12** | **China: Yunnan** | ***H.brasiliensis*** | **MG253879** | **MG253889** | **MG253899** | **MG253909** | **MG266134** | **MG266144** | **MG266154** | This study |
|  | **BS16116** | **China: Hainan** | ***H.brasiliensis*** | **MG253880** | **MG25390** | **MG253900** | **MG253910** | **MG266135** | **MG266145** | **MG266155** | This study |
|  | **GD1647** | **China: Guangdong** | ***H.brasiliensis*** | **MG253881** | **MG253891** | **MG253901** | **MG253911** | **MG266136** | **MG266146** | **MG266156** | This study |
| ***C. fructivorum*** | CBS 133125* | USA | *Vaccinium macrocarpon* | JX145145 | JX145196 | — | — | — | — | — | Doyle et al. 2013 |
| ***C. gloeosporioides*** | CBS 112999* | Italy | *Citrus sinensis* | JX010152 | JX010445 | JX009731 | JX009531 | JX010056 | JX009818 | JX010085 | Weir et al. 2012 |
|  | LC3382 | China | *Ca. sinensis,* | KJ955176 | KJ955323 | KJ954728 | KJ954450 | KJ954877 | — | KJ955026 | Liu et al. 2015 |
| ***C. grevilleae*** | CPC 15481* | Italy | *Grevillea sp.* | KC297078 | KC297102 | KC296963 | KC296941 | KC297010 | — | KC297033 | Liu et al. 2013 |
| ***C. henanense*** | LF238 * | China | *Ca. sinensis* | KJ955109 | KJ955257 | KJ954662 | KM023257 | KJ954810 | — | KJ954960 | Liu et al. 2015 |
| ***C. horii*** | MTCC 10841* | Japan | *Diospyros kaki* | GQ329690 | JX010450 | JX009604 | JX009438 | JQ329681 | JX009752 | JX010637 | Weir et al. 2012 |
| ***C. jiangxiense*** | LF687* | China | *Ca. sinensis,* | KJ955201 | KJ955348 | KJ954752 | KJ954471 | KJ954902 | — | KJ955901 | Liu et al. 2015 |
| ***C. kahawae subsp. ciggaro*** | ICMP 12952 | New Zealand | *Persea americana* | JX010214 | JX010426 | JX009648 | JX009431 | JX009971 | JX009757 | JX010126 | Weir et al. 2012 |
|  | ICMP 18539* | Australia | *Olea europaea* | JX010230 | JX010434 | JX009635 | JX009523 | JX009966 | JX009800 | JX010132 | Weir et al. 2012 |
| ***C. kahawae subsp. kahawae*** | ICMP 17816* | Kenya | *Coffea arabica* | JX010231 | JX010444 | JX009642 | JX009452 | JX010012 | JX009813 | JX010130 | Weir et al. 2012 |
|  | CBS 982.6 | Angola | *Coffea arabica* | JX010234 | JX010435 | JX009638 | JX009474 | JX010040 | JX009829 | JX010125 | Weir et al. 2012 |
| ***C. ledongense*** | **LD1680** | **China:Hainan** | ***H.brasiliensis*** | **MG242008** | **MG242010** | **MG242012** | **MG242014** | **MG242016** | **MG242018** | **MG242020** | This study |
|  | **LD1683**, **CGMCC 3.18888*** | **China:Hainan** | ***H.brasiliensis*** | **MG242009** | **MG242011** | **MG242013** | **MG242015** | **MG242017** | **MG242019** | **MG242021** | This study |
| ***C. musae*** | CBS 116870, MTCC1134* | USA | *Musa sp.* | JX010146 | HQ596280 | JX009742 | JX009433 | JX010050 | JX009815 | JX010103 | Su et al. 2011 |
| ***C. nupharicola*** | ICMP 18187* | USA | *Nuphar lutea* | JX010187 | JX010398 | JX009663 | JX009437 | JX009972 | JX009835 | JX010088 | Weir et al. 2012 |
| ***C. proteae*** | CPC 14859* | South Africa | *Protea sp.* | KC297079 | KC297101 | KC296960 | KC296940 | KC297009 | — | KC297032 | Liu et al. 2013 |
| ***C. psidii*** | ICMP 19120* | Italy | *Psidium sp.* | JX010219 | JX010443 | JX009743 | JX009515 | JX009967 | JX009901 | JX010133 | Weir et al. 2012 |
| ***C. queenslandicum*** | ICMP 1778* | Australia | *Carica papaya* | JX010276 | JX010414 | JX009691 | JX009447 | JX009934 | JX009899 | JX010104 | Weir et al. 2012 |
| ***C. rhexiae*** | CBS 133134* | USA | *Rhexia virginica* | JX145128 | JX145179 | — | — | — | — | — | Doyle et al. 2013 |
| ***C. salsolae*** | ICMP 19051* | Hungary | *Salsola tragus* | JX010242 | JX010403 | JX009696 | JX009562 | JX009916 | JX009863 | JX010093 | Weir et al. 2012 |
| ***C.siamense*** | CBS 130417* | Thailand | *Coffea arabica* | JX010171 | JX010404 | FJ917505 | FJ907423 | JX009924 | JX009865 | JX010094 | Phoulivong et al. 2009 |
|  | DAR 76934, | Australia | *Pistacia vera* | JX010270 | JX010391 | JX009707 | JX009535 | JX010002 | JX009798 | JX010080 | Weir et al. 2012 |
|  | **CJ1688** | **China:Hainan** | ***H.brasiliensis*** | **MG324013** | **MG324041** | **MG324181** | **MG324069** | **MG324097** | **MG324125** | **MG324153** | This study |
|  | **WN1679** | **China:Hainan** | ***H.brasiliensis*** | **MG324014** | **MG324042** | **MG324182** | **MG324070** | **MG324098** | **MG324126** | **MG324154** | This study |
|  | **CJ1690** | **China:Hainan** | ***H.brasiliensis*** | **MG324015** | **MG324043** | **MG324183** | **MG324071** | **MG324099** | **MG324127** | **MG324155** | This study |
|  | **GD1612** | **China:Guangdong** | ***H.brasiliensis*** | **MG324016** | **MG324044** | **MG324184** | **MG324072** | **MG324100** | **MG324128** | **MG324156** | This study |
|  | **WN1661** | **China:Hainan** | ***H.brasiliensis*** | **MG324017** | **MG324045** | **MG324185** | **MG324073** | **MG324101** | **MG324129** | **MG324157** | This study |
|  | **YNJH71** | **China: Yunnan** | ***H.brasiliensis*** | **MG324018** | **MG324046** | **MG324186** | **MG324074** | **MG324102** | **MG324130** | **MG324158** | This study |
|  | **GD1615** | **China:Guangdong** | ***H.brasiliensis*** | **MG324019** | **MG324047** | **MG324187** | **MG324075** | **MG324103** | **MG324131** | **MG324159** | This study |
|  | **CJ1698** | **China:Hainan** | ***H.brasiliensis*** | **MG324020** | **MG324048** | **MG324188** | **MG324076** | **MG324104** | **MG324132** | **MG324160** | This study |
|  | **DZ16047** | **China:Hainan** | ***H.brasiliensis*** | **MG324021** | **MG324049** | **MG324189** | **MG324077** | **MG324105** | **MG324133** | **MG324161** | This study |
|  | **GX1649** | **China:Guangxi** | ***H.brasiliensis*** | **MG324022** | **MG324050** | **MG324190** | **MG324078** | **MG324106** | **MG324134** | **MG324162** | This study |
|  | **BS16114** | **China:Hainan** | ***H.brasiliensis*** | **MG324023** | **MG324051** | **MG324191** | **MG324079** | **MG324107** | **MG324135** | **MG324163** | This study |
|  | **GD1635** | **China:Guangdong** | ***H.brasiliensis*** | **MG324024** | **MG324052** | **MG324192** | **MG324080** | **MG324108** | **MG324136** | **MG324164** | This study |
|  | **DZ16046** | **China:Hainan** | ***H.brasiliensis*** | **MG324025** | **MG324053** | **MG324193** | **MG324081** | **MG324109** | **MG324137** | **MG324165** | This study |
|  | **YNBD55** | **China: Yunnan** | ***H.brasiliensis*** | **MG324026** | **MG324054** | **MG324194** | **MG324082** | **MG324110** | **MG324138** | **MG324166** | This study |
|  | **BS16122** | **China:Hainan** | ***H.brasiliensis*** | **MG324027** | **MG324055** | **MG324195** | **MG324083** | **MG324111** | **MG324139** | **MG324167** | This study |
|  | **CJ16100** | **China:Hainan** | ***H.brasiliensis*** | **MG324028** | **MG324056** | **MG324196** | **MG324084** | **MG324112** | **MG324140** | **MG324168** | This study |
|  | **GD1636** | **China:Guangdong** | ***H.brasiliensis*** | **MG324029** | **MG324057** | **MG324197** | **MG324085** | **MG324113** | **MG324141** | **MG324169** | This study |
|  | **LGCg** | **China:Hainan** | ***H.brasiliensis*** | **MG324030** | **MG324058** | **MG324198** | **MG324086** | **MG324114** | **MG324142** | **MG324170** | This study |
|  | **WN16138** | **China:Hainan** | ***H.brasiliensis*** | **MG324031** | **MG324059** | **MG324199** | **MG324087** | **MG324115** | **MG324143** | **MG324171** | This study |
|  | **WN1671** | **China:Hainan** | ***H.brasiliensis*** | **MG324032** | **MG324060** | **MG324200** | **MG324088** | **MG324116** | **MG324144** | **MG324172** | This study |
|  | **YNMX101** | **China:Yunnan** | ***H.brasiliensis*** | **MG324033** | **MG324061** | **MG324201** | **MG324089** | **MG324117** | **MG324145** | **MG324173** | This study |
|  | **YNBD54** | **China:Yunnan** | ***H.brasiliensis*** | **MG324034** | **MG324062** | **MG324202** | **MG324090** | **MG324118** | **MG324146** | **MG324174** | This study |
|  | **YNJH74** | **China:Yunnan** | ***H.brasiliensis*** | **MG324035** | **MG324063** | **MG324203** | **MG324091** | **MG324119** | **MG324147** | **MG324175** | This study |
|  | **GD1602** | **China:Guangdong** | ***H.brasiliensis*** | **MG324036** | **MG324064** | **MG324204** | **MG324092** | **MG324120** | **MG324148** | **MG324176** | This study |
|  | **QZ16132** | **China:Hainan** | ***H.brasiliensis*** | **MG324037** | **MG324065** | **MG324205** | **MG324093** | **MG324121** | **MG324149** | **MG324177** | This study |
|  | **QZ8152** | **China:Hainan** | ***H.brasiliensis*** | **MG324038** | **MG324066** | **MG324206** | **MG324094** | **MG324122** | **MG324150** | **MG324178** | This study |
|  | **GX1650** | **China:Guangxi** | ***H.brasiliensis*** | **MG324039** | **MG324067** | **MG324207** | **MG324095** | **MG324123** | **MG324151** | **MG324179** | This study |
|  | **WN1665** | **China:Hainan** | ***H.brasiliensis*** | **MG324040** | **MG324068** | **MG324208** | **MG324096** | **MG324124** | **MG324152** | **MG324180** | This study |
| ***C.siamense(syn. C.hymenocallidis)*** | CBS 125378, | China | *Hymenocallis americana* | JX010278 | JX010410 | JX009709 | JX009441 | JX010019 | GQ856370 | JX010100 | Weir et al. 2012 |
| ***C.siamense(syn. C.jasmini-sambac)*** | CBS 130420, | Vietnam | *Jasminum sambac* | HM131511 | JX010415 | JX009713 | HM131507 | HM131497 | JX009895 | JX010105 | Weir et al. 2012 |
| ***C.siamense (syn. C. murrayae)*** | GZAAS 5.09506 | China | *Murraya sp.* | JQ247633 | JQ247644 | JQ247596 | JQ247657 | JQ247609 | — | JQ247621 | Weir et al. 2012 |
| ***C. syzygicola*** | MFLUCC100624 |  | *Syzygium samarangense* | KF242094 | KF254880 | KF254859 | KF157801 | KF242156 | — | KF242125 | Udayanga et al.2013 |
| ***C. temperatum*** | CBS 133122* | USA | *Vaccinium macrocarpon* | JX145159 | JX145211 | — | — | — | — | — | Doyle et al. 2013 |
| ***C. theobromicola*** | ICMP 18649* | Panama | *Theobroma cacao* | JX010294 | JX010447 | JX009591 | JX009444 | JX010006 | JX009869 | JX010139 | Weir et al. 2012 |
| ***C. ti*** | ICMP 4832* | New Zealand | *Cordyline sp.* | JX010269 | JX010442 | JX009649 | JX009520 | JX009952 | JX009898 | JX010123 | Weir et al. 2012 |
| ***C. tropicale*** | MTCC 11371* | Panama | *Theobroma cacao* | JX010264 | JX010407 | JX009719 | JX009489 | JX010007 | JX009870 | JX010097 | Weir et al. 2012 |
| ***C. viniferum*** | GZAAS5.08601* | China | *Vitis vinifera* | JN412804 | — | JQ309639 | JN412795 | JN412798 | — | JN412787 | Peng et al. 2013 |
| ***C. xanthorrhoeae*** | CBS 127831* | Australia | *Xanthorrhoea preissii* | JX010261 | JX010448 | JX009653 | JX009478 | JX009927 | JX009823 | JX010138 | Shivas et al. 1998; Weir et al. 2012 |

ex-holotype or ex-epitype cultures. Strains studied in this report are in **bold** font.

**Table S 2.** Strains of the *C.* *acutatum* s.l. species studied in this report with details on host, location, and GenBank accessions of the sequences generated.

| **Species** | **Strains** | **Locality** | **Host** | **GenBank accessions** | | | | | **Reference** |
| --- | --- | --- | --- | --- | --- | --- | --- | --- | --- |
|  |  |  |  | **ITS** | **TUB2** | **ACT** | **GAPDH** | **CHS-1** |  |
| ***C. acerbum*** | CBS128530, PRJ 1199.3* | New Zealand | Malus domestica | JQ948459 | JQ950110 | JQ949780 | JQ948790 | JQ949120 | Damm et al. 2012 |
| ***C. acutatum*** | CBS 144.29 | Sri Lanka | *Capsicum annuum* | JQ948401 | JQ950052 | JQ949722 | JQ948732 | JQ949062 | Damm et al. 2012 |
|  | CBS112996, STE-U 5292* | Australia | *Carica papaya* | JQ005776 | JQ005860 | JQ005839 | JQ948677 | JQ005797 | Damm et al. 2012 |
| ***C. australe*** | CBS116478, HKUCC 2616* | South Africa | Trachycarpus fortunei | JQ948455 | JQ950106 | JQ949776 | JQ948786 | JQ949116 | Damm et al. 2012 |
| ***C. bannaense*** | **YNBD42** | **China:Yunnan** | **Hevea brasiliensis** | **MG209637** | **MG209659** | **MG242001** | **MG242005** | **MG241995** | **This study** |
|  | **YNML52,** **CGMCC 3.18887*** | **China:Yunnan** | **H.brasiliensis** | **MG209638** | **MG209660** | **MG242002** | **MG242006** | **MG241996** | **This study** |
|  | **YNWD31** | **China:Yunnan** | **H.brasiliensis** | **MG209639** | **MG209661** | **MG242003** | **MG242007** | **MG241997** | **This study** |
| ***C. brisbanense*** | CBS292.67, DPI 11711* | Australia | Capsicum annuum | JQ948291 | JQ949942 | JQ949612 | JQ948621 | JQ948952 | Damm et al. 2012 |
| ***C. cairnsense*** | BRIP 63641 | Australia | C. annuum | KU923671 | KU923687 | KU923715 | KU923703 | KU923709 | De Silva et al. 2017 |
|  | BRIP 63642* | Australia | C. annuum | KU923672 | KU923688 | KU923716 | KU923704 | KU923710 | De Silva et al. 2017 |
| ***C. chrysanthemi*** | IMI364540, CPC 18930 | China | Chrysanthemum coronarium, | JQ948273 | JQ949924 | JQ949594 | JQ948603 | JQ948934 | Damm et al. 2012 |
| ***C. cosmi*** | CBS853.73, PD 73/856* | Netherlands | Cosmos sp. | JQ948274 | JQ949925 | JQ949595 | JQ948604 | JQ948935 | Damm et al. 2012 |
| ***C. costaricense*** | CBS 330.75* | Costa Rica | Coffea arabica | JQ948180 | JQ949831 | JQ949501 | JQ948510 | JQ948841 | Damm et al. 2012 |
| ***C. cuscutae*** | IMI304802, CPC 18873* | Dominica | Cuscuta sp. | JQ948195 | JQ949846 | JQ949516 | JQ948525 | JQ948856 | Damm et al. 2012 |
| ***C. fioriniae*** | CBS127601, BRIP 28761***** | Australia | Mangifera indica | JQ948311 | JQ949962 | JQ949632 | JQ948641 | JQ948972 | Damm et al. 2012 |
|  | CBS129947, CR46, RB022 | Portugal | Vitis vinifera | JQ948343 | JQ949994 | JQ949664 | JQ948673 | JQ949004 | Damm et al. 2012 |
| ***C. godetiae*** | CBS 133.44* | Denmark | Clarkia hybrida | JQ948402 | JQ950053 | JQ949723 | JQ948733 | JQ949063 | Damm et al. 2012 |
| ***C. guajavae*** | IMI350839, CPC 18893* | India | Psidium guajava | JQ948270 | JQ949921 | JQ949591 | JQ948600 | JQ948931 | Damm et al. 2012 |
| ***C. indonesiense*** | CBS127551, CPC 14986* | Indonesia | Eucalyptus sp. | JQ948288 | JQ949939 | JQ949609 | JQ948618 | JQ948949 | Damm et al. 2012 |
| ***C. johnstonii*** | CBS128532, ICMP12926, PRJ 1139.3* | New Zealand | Solanum lycopersicum, | JQ948444 | JQ950095 | JQ949765 | JQ948775 | JQ949105 | Damm et al. 2012 |
| ***C. kinghornii*** | CBS 198.35* | UK | Phormium sp. | JQ948454 | JQ950105 | JQ949775 | JQ948785 | JQ949115 | Damm et al. 2012 |
| ***C. laticiphilum*** | CBS112989, IMI383015, STE-U 5303* | India | Hevea brasiliensis | JQ948289 | JQ949940 | JQ949610 | JQ948619 | JQ948950 | Damm et al. 2012 |
|  | CBS 129827, | Colombia | Hevea brasiliensis, | JQ948290 | JQ949941 | JQ949611 | JQ948620 | JQ948951 | Damm et al. 2012 |
| ***C. limetticola*** | CBS 114.14* | USA, Florida | Citrus aurantifolia, | JQ948193 | JQ949844 | JQ949514 | JQ948523 | JQ948854 | Damm et al. 2012 |
| ***C. lupini*** | CBS 109225, BBA 70884* | Ukraine | Lupinus albus | JQ948155 | JQ949806 | JQ949476 | JQ948485 | JQ948816 | Damm et al. 2012 |
| ***C. melonis*** | CBS 159.84* | Brazil | Cucumis melo | JQ948194 | JQ949845 | JQ949515 | JQ948524 | JQ948855 | Damm et al. 2012 |
| ***C. nymphaeae*** | CBS 129929 | USA | Fragaria × ananassa | JQ948229 | JQ949880 | JQ949550 | JQ948559 | JQ948890 | Damm et al. 2012 |
|  | CBS 515.78* | Netherlands | Nymphaea alba | JQ948197 | JQ949848 | JQ949518 | JQ948527 | JQ948858 | Damm et al. 2012 |
| ***C. orchidophilum*** | CBS 632.80* | USA | Dendrobium sp. | JQ948151 | JQ949802 | JQ949472 | JQ948481 | JQ948812 | Damm et al. 2012 |
| ***C. paxtonii*** | IMI165753, CPC 18868* | Saint Lucia | Musa sp. | JQ948285 | JQ949936 | JQ949606 | JQ948615 | JQ948946 | Damm et al. 2012 |
| ***C. phormii*** | CBS118194, AR 3546* | Germany | Phormium sp. | JQ948446 | JQ950097 | JQ949767 | JQ948777 | JQ949107 | Damm et al. 2012 |
| ***C. pseudoacutatum*** | CBS 436.77* | Chile | Pinus radiata | JQ948480 | JQ950131 | JQ949801 | JQ948811 | JQ949141 | Damm et al. 2012 |
| ***C. pyricola*** | CBS128531, ICMP12924, PRJ 977.1* | New Zealand | Pyrus communis | JQ948445 | JQ950096 | JQ949766 | JQ948776 | JQ949106 | Damm et al. 2012 |
| ***C. rhombiforme*** | CBS129953, RB011* | Portugal | Olea europaea | JQ948457 | JQ950108 | JQ949778 | JQ948788 | JQ949118 | Damm et al. 2012 |
| ***C. salicis*** | CBS 607.94* | Netherlands | Salix sp. | JQ948460 | JQ950111 | JQ949781 | JQ948791 | JQ949121 | Damm et al. 2012 |
| ***C. scovillei*** | CBS126529, BBA 70349* | Indonesia | Capsicum sp. | JQ948267 | JQ949918 | JQ949588 | JQ948597 | JQ948928 | Damm et al. 2012 |
| ***C. simmondsii*** | CBS122122, BRIP 28519* | Carica papaya | Australia | JQ948276 | JQ949927 | JQ949597 | JQ948606 | JQ948937 | Damm et al. 2012 |
| ***C. sloanei*** | IMI364297, CPC 18929* | Malaysia | Theobroma cacao | JQ948287 | JQ949938 | JQ949608 | JQ948617 | JQ948948 | Damm et al. 2012 |
| ***C australisinense*** | **BS16117** | **China:Hainan** | **H.brasiliensis** | **MG209618** | **MG209640** | **MG241942** | **MG241958** | **MG241976** | **This study** |
|  | **BS16111** | **China:Hainan** | **H. brasiliensis** | **MG209619** | **MG209641** | **MG241943** | **MG241959** | **MG241977** | **This study** |
|  | **WN1670** | **China:Hainan** | **H.brasiliensis** | **MG209620** | **MG209642** | **MG241944** | **MG241960** | **MG241978** | **This study** |
|  | **GX1654** | **China:Guangxi** | **H.brasiliensis** | **MG209621** | **MG209643** | **MG241945** | **MG242004** | **MG241979** | **This study** |
|  | **WN1678** | **China:Hainan** | **H.brasiliensis** | **MG209622** | **MG209644** | **MG241946** | **MG241961** | **MG241980** | **This study** |
|  | **GX1655, CGMCC 3.18886*** | **China:Guangxi** | **H.brasiliensis** | **MG209623** | **MG209645** | **MG241947** | **MG241962** | **MG241981** | **This study** |
|  | **BS16105** | **China:Hainan** | **H.brasiliensis** | **MG209624** | **MG209646** | **MG241998** | **MG241963** | **MG241982** | **This study** |
|  | **BS16107** | **China:Hainan** | **H.brasiliensis** | **MG209625** | **MG209647** | **MG241999** | **MG241964** | **MG241983** | **This study** |
|  | **DZ16042** | **China:Hainan** | **H.brasiliensis** | **MG209626** | **MG209648** | **MG242000** | **MG241965** | **MG241984** | **This study** |
|  | **QZ16129** | **China:Hainan** | **H.brasiliensis** | **MG209627** | **MG209649** | **MG241948** | **MG241966** | **MG241985** | **This study** |
|  | **QZ16131** | **China:Hainan** | **H.brasiliensis** | **MG209628** | **MG209650** | **MG241949** | **MG241967** | **MG241986** | **This study** |
|  | **GX1652** | **China:Guangxi** | **H.brasiliensis** | **MG209629** | **MG209651** | **MG241950** | **MG241968** | **MG241987** | **This study** |
|  | **QZ16130** | **China:Hainan** | **H.brasiliensis** | **MG209630** | **MG209652** | **MG241951** | **MG241969** | **MG241988** | **This study** |
|  | **GD1607** | **China:Guangdong** | **H.brasiliensis** | **MG209631** | **MG209653** | **MG241952** | **MG241970** | **MG241989** | **This study** |
|  | **LD1687** | **China:Hainan** | **H.brasiliensis** | **MG209632** | **MG209654** | **MG241953** | **MG241971** | **MG241990** | **This study** |
|  | **LD1682** | **China:Hainan** | **H.brasiliensis** | **MG209633** | **MG209655** | **MG241954** | **MG241972** | **MG241991** | **This study** |
|  | **GD1609** | **China:Guangdong** | **H.brasiliensis** | **MG209634** | **MG209656** | **MG241955** | **MG241973** | **MG241992** | **This study** |
|  | **YNJP162** | **China:Yunnan** | **H.brasiliensis** | **MG209635** | **MG209657** | **MG241956** | **MG241974** | **MG241993** | **This study** |
|  | **GD1610** | **China:Guangdong** | **H.brasiliensis** | **MG209636** | **MG209658** | **MG241957** | **MG241975** | **MG241994** | **This study** |
| ***C. tamarilloi*** | CBS129814, T.A.6* | Colombia | Solanum betaceum | JQ948184 | JQ949835 | JQ949505 | JQ948514 | JQ948845 | Damm et al. 2012 |
| ***C. walleri*** | CBS125472, BMT(HL)19* | Vietnam | Coffea sp. | JQ948275 | JQ949926 | JQ949596 | JQ948605 | JQ948936 | Damm et al. 2012 |

* ex-holotype or ex-epitype cultures. Strains studied in this report are in **bold** font.
